# Quantification of Kidney Inflammation Using Nanobubble-mediated Contrast Enhanced Ultrasound

**DOI:** 10.7150/ntno.126443

**Published:** 2026-02-11

**Authors:** Niloufar Rostam Shirazi, Xiaolin He, Dana Dranka, Omar Falou, Eno Hysi, Agata A. Exner, Darren Yuen, Michael C. Kolios

**Affiliations:** 1 Department of Physics, Faculty of Science, Toronto Metropolitan University, Toronto, Ontario, Canada.; 2 Institute for Biomedical Engineering, Science and Technology, a partnership between Toronto Metropolitan University and St. Michael's Hospital, Toronto, Ontario, Canada.; 3 Keenan Research Centre for Biomedical Science, Li Ka Shing Knowledge Institute, St. Michael's Hospital, Unity Health Toronto, Toronto, Ontario, Canada.; 4 Department of Radiology, Case Western Reserve University School of Medicine, Cleveland, OH, USA.; 5 Department of Medical Biophysics, Temerty Faculty of Medicine, University of Toronto, Toronto, Ontario, Canada.; 6 Center for Imaging Research, Case Western Reserve University, Cleveland, OH, USA.; 7 Department of Biomedical Engineering, Case School of Engineering, Case Western Reserve University, Cleveland, OH, USA.; 8 Department of Medicine, Temerty Faculty of Medicine, University of Toronto, Toronto, Ontario, Canada.

## Abstract

Kidney inflammation is a central driver of acute kidney injury (AKI) and its progression to chronic kidney disease (CKD). While several imaging and biomarker-based approaches are under development, clinically validated non-invasive methods to directly quantify renal inflammation remain limited. This study introduces a novel approach using contrast-enhanced ultrasound (CEUS) with Cy5-labeled nanobubbles (NBs) to address this critical knowledge gap. Using a murine ischemia-reperfusion injury (IRI) model, CEUS imaging enabled real-time visualization of inflammation-induced changes in kidney perfusion and vascular integrity. Parametric analyses of non-linear imaging revealed delayed time-to-peak (TTP) and increased area under the falling curve (AUfC) in IRI kidneys, suggesting impaired microvascular perfusion and NB retention. Decorrelation time (DT) mapping further identified prolonged NB retention in the IRI group, indicating increased capillary permeability and NB extravasation. These findings correlated with histological and immunofluorescent analyses, which confirmed the presence of tubular injury, extravascular Cy5 signal localization, and increased neutrophil infiltration in inflamed kidney tissues. This study is the first to establish CEUS with NBs as a non-invasive, quantitative method for measuring kidney inflammation. With strong correlations between imaging metrics and histologic injury scores, this technology provides an accessible and non-invasive tool for monitoring renal inflammation and reducing reliance on invasive renal biopsies.

## Introduction

Acute kidney injury (AKI) is a common complication, affecting up to 20% of hospitalized patients, and is associated with 30% of in-hospital mortalities 1. Inflammation is central to AKI pathophysiology, leading to endothelial dysfunction, increased vascular permeability, and immune cell infiltration. These microvascular changes often occur early and precede measurable declines in renal function.

Renal biopsy remains the gold standard method for assessing inflammation in AKI. However, it is invasive, painful, and prone to sampling bias, since it typically captures less than 1% of the kidney parenchyma 2,3. Extensive efforts to develop non-invasive tools to replace the kidney biopsy have been undertaken over the years, but to date they have largely failed to directly or quantitatively assess renal inflammation 4. For example, blood- and urine-based biomarkers such as neutrophil gelatinase-associated lipocalin (NGAL) 5-8, kidney injury molecule-1 (KIM-1) 5, interleukin-18 (IL-18) 6 and protein C 5,9 have been widely studied as markers of tubular injury and immune activation. However, these markers are also influenced by systemic factors, and have unfortunately not demonstrated adequate specificity to reliably quantify renal inflammation 10.

Magnetic resonance imaging (MRI) has been used to evaluate post-ischemic renal changes such as reduced perfusion, edema, and interstitial inflammation. Techniques including diffusion-weighted imaging, and dynamic contrast-enhanced MRI detect perfusion deficits, restricted diffusivity, and increased permeability after ischemia-reperfusion injury (IRI) 11,12. These imaging biomarkers correlate with inflammatory cytokine levels and have been associated with fibrosis and renal function decline 12,13. Similarly, positron emission tomography (PET) has been used to detect markers of renal inflammation after IRI, such as immune cell infiltration 14. However, the cost, limited accessibility, reliance on contrast agents, and harmful radiation (in the case of PET) have limited the use of either of these modalities for routine monitoring.

Doppler ultrasound (US) has been used to assess kidneys following ischemic AKI by measuring blood flow in the large renal vessels 15. The renal resistive index (RI), a hemodynamic marker, correlates with AKI severity and outcomes 16, however, it is influenced by systemic factors (e.g., blood pressure, vascular stiffness) and is not a direct measure of inflammation 17. Studies have also shown that Doppler changes usually manifest only after significant renal damage has occurred, reducing its utility for early inflammation detection 16. Thus, it lacks the sensitivity and specificity required to directly and quantitatively assess kidney inflammation 17.

Gas-filled microbubbles (MBs) with typical size distribution of 1-10 μm 18,19 are widely used as contrast agents in a technique, called microbubble contrast-enhanced US (MB-CEUS) to quantitatively assess renal perfusion 20. In IRI models, MB-CEUS has demonstrated reduced blood flow in the ischemic cortex and medulla 21. Although an important advance, these findings measure changes in flow rather than renal inflammation, like Doppler US. To address this, MBs have been modified to target adhesion molecules such as P-selectin or VCAM-1, enabling detection of endothelial activation within hours of injury 22. Although promising, endothelial activation alone does not provide a direct measure of tissue inflammation 23.

This work presents a new approach to spatially map kidney inflammation using nanobubble (NB)-CEUS. Due to their nanoscale size, NBs can exit the vasculature through endothelial gaps that develop in the hyperpermeable blood vessels found only in inflamed and malignant tissue 24-26. These gaps can range from approximately 380-780 nm in inflamed and neoplastic tissues, whereas endothelial junctions in healthy organs are tightly sealed, with pore sizes typically less than 7 nm, effectively preventing NB extravasation 27. Since NBs can be used like MBs to assess perfusion, their unique ability to extravasate into inflamed tissue also allows for the quantification of vascular permeability and organ injury. In recent studies, we have demonstrated that NB-CEUS can be used to measure microvascular leakage in both tumors and inflammation of the pancreas 28, offering patterns of contrast enhancement that MBs cannot achieve 27,29,30.

Here, we demonstrate that NB-CEUS can distinguish physiologically relevant differences in perfusion and microvascular leak between IRI-damaged and healthy kidneys. Using spatially resolved parametric maps, we assessed NB inflow (perfusion) and outflow (capillary leak). This is the first demonstration of NB-CEUS as a simple, non-invasive method to evaluate renal microvascular perfusion and permeability during inflammatory injury.

## Methods

### Animal Model of Ischemia Reperfusion Injury

All animal studies were approved by the St. Michael's Hospital Animal Ethics Committee and conformed to the Canadian Council on Animal Care guidelines. Ten male BALB/C mice (8 weeks, 22-25 grams, Charles River Laboratories, Laval, Quebec, Canada) underwent bilateral IRI (n = 6) for 25 minutes according to our previously published protocol 31 or sham surgery (n = 4). Briefly, mice were anesthetized with 2.5% isoflurane (Fresenius Kabi, Toronto, ON, Canada) in 100% oxygen (0.5 L/min) and maintained on a heating pad. A midline laparotomy was performed, and ischemia was induced by clamping both renal arteries and veins with non-traumatic microvascular clamps for 25 minutes. Reperfusion was visually confirmed after clamp removal, followed by 0.02 mL slow-release buprenorphine (Chiron Compounding Pharmacy Inc., Guelph, ON, Canada). Mice recovered in a warmed area before being returned to heated cages for 24 hours.

### Fluorescent NB Preparations and Size Measurement

NBs conjugated to Cyanine5 (Cy5, excitation 649 nm, emission 670 nm) were used for imaging to reduce confounding signals from renal autofluorescence. Cy5-NBs were prepared as per our published protocol 32-34. Briefly, Cy5-labeled NBs were prepared by evaporating chloroform from 100 µL DSPE-PEG-Cy5 (1 mg/mL; Biopharma PEG Scientific Inc., Watertown, MA, USA) in a water bath at 80 °C. A lipid mixture of DBPC, DPPE, and DPPA (Avanti®, Sigma-Aldrich, St. Louis, MO, USA) with propylene glycol (Fisher Scientific, Portsmouth, NH, USA) was added and heated until dissolved. A glycerol (Sigma-Aldrich)/PBS (Wisent, Saint-Jean-Baptiste, QC, Canada) solution was then combined with the lipids at 80 °C and sonicated (Branson Ultrasonics Corp., Brookfield, CT, USA) until transparent. The mixture was sonicated again for 10 min at room temperature and stored at 4 °C in 3 mL headspace vials. To activate NBs, vials were degassed, filled with C₃F₈ gas, and agitated using a VialMix shaker (Bristol Myers Squibb, New York, NY, USA). NBs were isolated by centrifugation at 50 rcf for 5 min (MXIE TD5A-WS, Beckman Coulter Canada Inc., Mississauga, ON, Canada) and extracted with a 5-mm trimmed needle cap to collect only the NB-rich layer.

Extracted Cy5-labeled NBs were diluted 1:1000 in PBS, and their size and concentration measured with the Archimedes Particle Metrology System (Malvern Panalytical, MA, USA). Archimedes uses resonant mass measurement (RMM) to detect, size, and count particles from 50 nm to 5 µm. RMM is a well-established technique for quantifying both buoyant and non-buoyant particles 34.

### Contrast Enhanced Ultrasound (CEUS) Imaging

CEUS imaging with Cy5-labeled NBs was performed 24 hours post-surgery to capture the early inflammatory period following IRI 35-37.

Four sham and six BALB/C mice were anesthetized with 2.5% isoflurane in oxygen (0.5 L/min). Fur over the kidney was removed, and mice were positioned prone on a heated platform (42 °C). Body temperature was maintained at 36.6-37 °C using a rectal probe. Each mouse received 200 µL Cy5-labeled NBs (350 nm, 6×10¹⁰/mL) via tail vein injection with a 27G butterfly catheter (SAI Infusion Technologies, IL, USA) over 30 seconds Supplementary Figure 1.

The NB concentration used in this study, while higher than that typically reported for MB-based CEUS or US localization microscopy, was selected to account for the reduced per-particle echogenicity of NBs. Due to their smaller size, individual NBs generate weaker nonlinear signals than MBs, necessitating higher particle concentrations to achieve sufficient contrast-to-noise for nonlinear CEUS imaging 33,38. Importantly, this study does not rely on sparse single-bubble localization like US localization microscopy but instead analyzes ensemble contrast dynamics and temporal decorrelation, which are not degraded at higher particle densities 28,38.

CEUS imaging was performed on a VisualSonics VEVO 2100 system (FujiFilm-VisualSonics, Toronto, ON) using an 18-MHz linear array transducer at 4% power (MI 0.39; peak negative pressure 1.76 MPa 39. Each mouse underwent six continuous imaging acquisitions, with each acquisition capturing 1000 co-registered, B-mode, and contrast imaging frames at a rate of 5 frames per second, for a total scan duration of 20 minutes. These acquisitions are labelled Aq 1 to Aq 6. Two IRI animals (IRI 1 and IRI 6) succumbed at the end of acquisition 5, but both were included in the analysis up to their respective time points.

Immediately after the imaging, mice were euthanized, and a nephrectomy was performed. Each kidney was divided into two halves, with one half preserved in Tissue-Tek optimal cutting temperature (OCT) compound (Sakura Finetek, USA), and the other half fixed in formalin (Sigma- Aldrich, MO, USA) and embedded in paraffin.

### Histological Staining of Tubular Injury and Neutrophil Infiltration

To quantify the degree of tubular injury, 5 µm thick formalin-fixed, paraffin-embedded kidney sections were stained with hematoxylin and eosin (H&E) using a Leica Autostainer XL (Leica Biosystems, ON, Canada). Tubular injury was then assessed using a previously published semi-quantitative scoring system that analyzes features such as cellular necrosis, loss of the brush border, cast formation, and signs of tubular dilation and atrophy 40. The scoring criteria were as follows: a score of 1 indicated that 0-10% of the tubules were affected, 2 signified involvement of 10-25% of tubules, 3 represented 26-50%, 4 indicated 51%-75%, and 5 indicated > 75% tubular involvement 40. For each kidney section, three fields from the outer layer of the medulla were examined to determine the average tubular injury scores.

Immunohistochemistry was performed on 5-µm formalin-fixed, paraffin-embedded kidney sections using anti-GR1 (Ly-6G/Ly-6C) antibody clone RB6-8C5 (1:200; Cat# 14-5931-82, Thermo Fisher Scientific, Waltham, MA) to assess neutrophil infiltration, a hallmark of early acute inflammation. Five non-overlapping ROIs were analyzed per section. In FIJI ImageJ, images were converted to 8-bit grayscale, and DAB signal was isolated with the Color Deconvolution 'H DAB' vector. GR1-positive cells were thresholded and counted with “Analyze Particles,” and mean neutrophils/µm² calculated per kidney. In parallel, macrophages were detected with anti-F4/80 BM8 monoclonal antibody (1:2000; Cat# MCA487, Bio-Rad, Mississauga, ON, Canada). Images were acquired on an Olympus CX33 light microscope (Evident Scientific, Tokyo, Japan).

### Histological Staining of Endothelial Cells

Seven-µm OCT-embedded kidney sections were stained with anti-CD31 (1:100; Cat# 550274, BD Pharmingen, San Diego, CA) to label endothelial cells, followed by DAPI counterstaining. The primary anti-CD31 antibody was detected with a Purified Rat Anti-Mouse Alexa Fluor 488 secondary antibody (1:200; Cat# A11006, Invitrogen, Carlsbad, CA). Three to four non-overlapping 63× images of the outer medulla were acquired using a Quorum Spinning Disc confocal microscope (Quorum Technologies Inc., Puslinch, ON, Canada). To assess Cy5 localization, ImageJ was used to generate binary vessel masks from the CD31 channel and their inverted counterparts. These were overlaid with the Cy5 channel using the “AND” operator to quantify intravascular versus extravascular Cy5 signal.

### CEUS Image Analysis

#### Kidney Auto Segmentation

To reduce motion artifacts, kidneys were segmented from baseline B-mode images prior to NB injection. Segmentation used a pretrained DeepLabv3+ model (MATLAB's Deep Learning Toolbox (MATLAB, Natick, MA) fine-tuned on a custom CEUS kidney dataset with expert-annotated masks. Images were resized to 512×512 pixels for training. The model achieved Dice coefficients of 91.5% for kidney and 99.0% for background on a held-out test set, confirming accurate and reliable segmentation.

#### Time Intensity Curve and Parametric Mapping

After NB injection, time-intensity curves (TICs) of the non-linear CEUS signal were analyzed to track NB passage through the kidney 41. TIC parameters were calculated for the whole kidney and on a per-pixel basis, allowing localized analysis at the resolution of the imaging system. Parametric maps of time-to-peak (TTP), area under the curve (AUC), rising curve (AUrC), falling curve (AUfC), and peak intensity (PI) were generated for both IRI and sham groups.

#### Haralick Texture Features

Haralick features are statistical descriptors derived from the gray-level co-occurrence matrix (GLCM) that quantify textural qualities such as coarseness or smoothness 42. In this study, four GLCM-based features were applied to parametric maps: energy, reflecting uniformity and image homogeneity; contrast, quantifying intensity differences between neighboring pixels; correlation, assessing spatial organization of gray levels; and homogeneity, measuring the dominance of smooth textures 43. Analysis of these features enabled identification of more subtle differences between sham and IRI kidneys that are not readily apparent to the human eye.

#### Decorrelation Time (DT) Mapping

DT maps were calculated to assess the rate of temporal changes in NB contrast signal in each kidney, using previously published methods 44. Briefly, pixel-level TICs were normalized using z-scores, and the autocorrelation function was calculated for each pixel to determine the time at which the correlation dropped to a threshold of 0.5. This process was iterated across all spatial locations within the regions of interest, generating comprehensive DT maps 44. Echo decorrelation techniques distinguish fast-moving intravascular agents from slower or stationary extravascular agents. The resulting maps offer spatially resolved insights into NB dynamics, enabling localization and comparison of extravasated NBs between IRI and sham kidneys.

#### Statistical Analysis

Statistical analysis was performed using JMP Pro software (SAS Institute Inc., Cary, NC). Welch's *t*-test was used for comparisons between sham and IRI groups. Effect sizes were quantified using Glass's Δ to reflect differences in variability between groups. For correlation analyses between imaging parameters and histological scores, Pearson's rank correlation coefficient (r) was computed. P < 0.05 was considered significant.

## Results

### NB size distribution

Supplementary Figure 2A depicts the size distribution of Cy5 NBs with an average diameter of 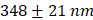. The total concentration of all NBs was 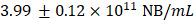. Cy5 NBs were then diluted to a concentration of
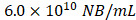
before injection into each mouse (total volume = 200μL). This concentration was determined from prior unpublished optimization studies as the highest that could be used without inducing shadowing artifacts in the US images.

### CEUS Quantification Signal Enhancement

**Figure 1:**
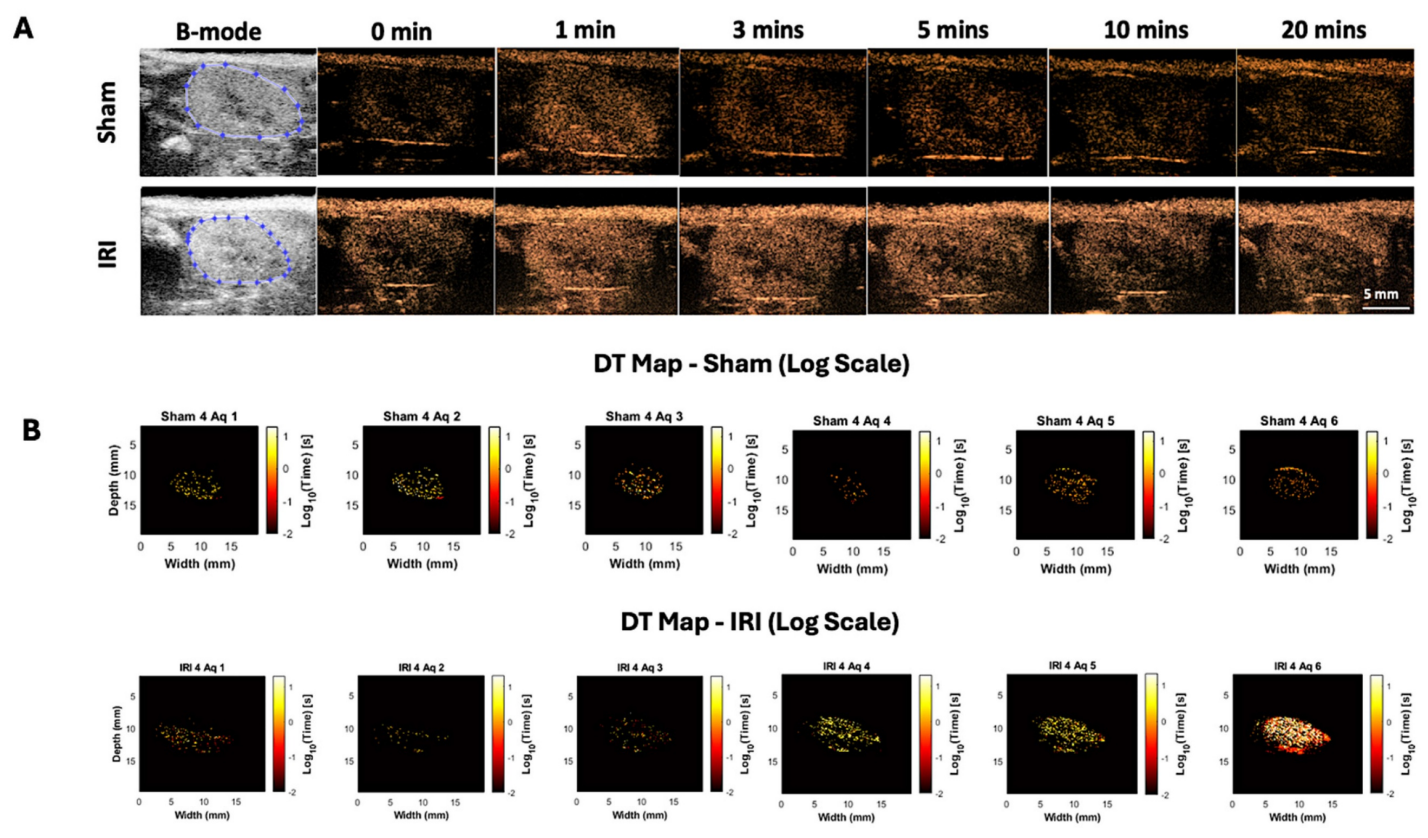

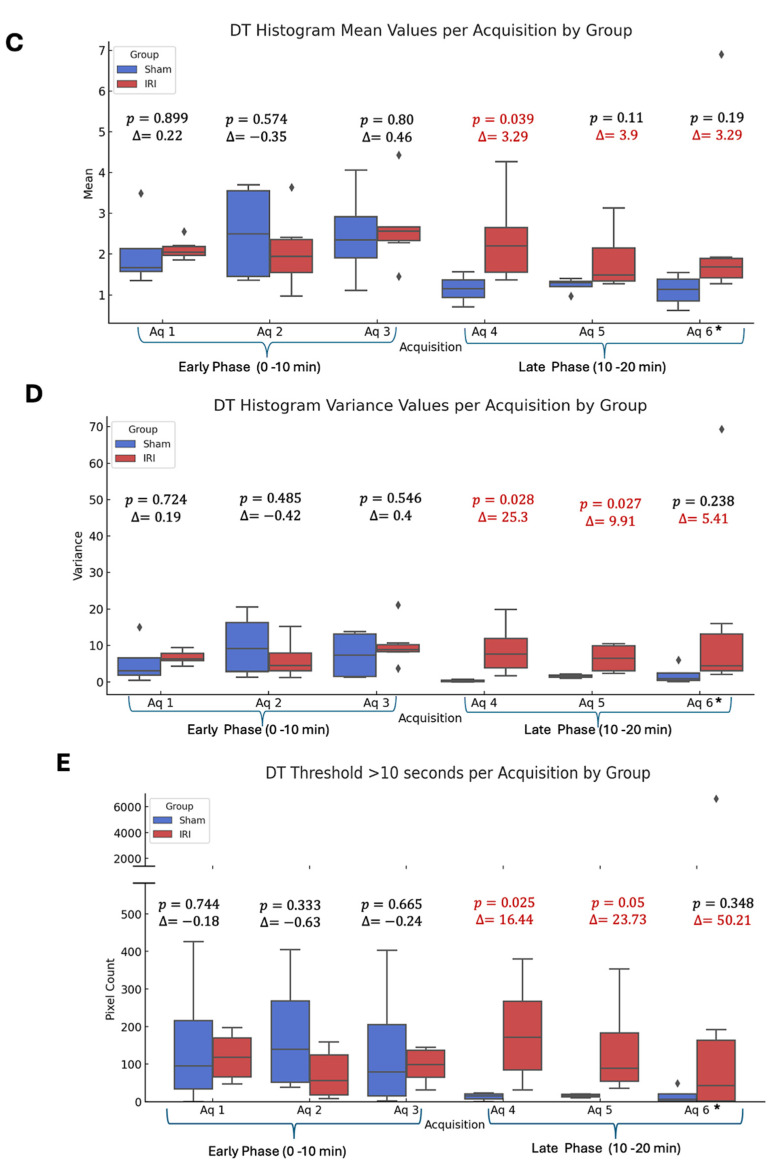
** Spatial and temporal distribution of log-transformed decorrelation time (DT) values in sham and IRI kidneys.** (A) B-mode baseline images display the anatomical kidney outline prior to NB injection. CEUS images acquired at sequential time points (0, 1, 3, 5, 10, and 20 minutes post-injection) show a rapid signal enhancement within the kidney regions, particularly during the early wash-in phase (0-3 minutes), with an average increase of ~12 dB, reflecting (B) Log-scaled DT maps of a representative sham and IRI kidney across six acquisitions. Each map shows the spatial distribution of log₁₀-transformed DT values. Acquisitions (Aq 1-6) were collected at 3.33-minute intervals during the NB wash-in and washout phase. (C) Mean DT values per acquisition for all sham and IRI kidneys. (D) Spatial variance of pixel-wise DT values within individual kidneys per acquisition, reflecting intra-animal heterogeneity (patchiness) of nanobubble retention rather than variability between animals. (E) Pixel counts exceeding a DT threshold of 10 seconds per acquisition. A split y-axis is used in (E) to display both lower and higher value ranges. p-values and Glass's Δ (IRI vs. Sham) for each acquisition are shown. Asterisk in acquisition 6 indicates that data are available for *n = 8* IRI animals at this time point, while *n = 2* IRI animals died during the imaging after acquisition 5 (and therefore do not have data for acquisition 6).

To evaluate the potential of NB-CEUS for detecting renal inflammation, sham and IRI mice underwent CEUS before and after Cy5-NB injection. Baseline B-mode images delineated the kidney outline in both groups (Figure 1A). Following injection, contrast intensity increased by ~12 dB within the first 3 minutes, reflecting NB inflow, and gradually declined between 5-20 minutes, consistent with washout. CEUS also resolved anatomical features, with the medulla consistently enhancing less than the cortex.

To objectively assess differences in contrast enhancement dynamics between sham and IRI kidneys, we quantified average signal amplitude (a.u.) over time (minutes) to generate whole-kidney TICs Supplementary Figure 2B). Although renal perfusion is known to be reduced early after IRI (45, peak NB-CEUS signal intensity was comparable between sham and IRI kidneys, suggesting similar initial NB accumulation. IRI kidneys, however, trended toward delayed inflow and impaired perfusion, reflected by prolonged TTP and reduced AUrC. Because NBs were expected to persist in inflamed IRI kidneys, we examined washout kinetics. Area under the falling curve (AUfC) was higher in IRI kidneys than in shams Supplementary Figure 2C), indicating delayed NB clearance from injured tissue.

Although none of these differences reached statistical significance (Supplementary Figure 2C), the trends pointed toward altered perfusion and retention dynamics in the injured kidneys. However, the overall variability within each group led to considerable overlap across metrics, suggesting that mean whole kidney-based measurements may not be very useful given the spatial heterogeneity of contrast dynamics following IRI.

### Decorrelation Time (DT) Maps

Whole-kidney TIC analysis suggested altered NB kinetics in IRI kidneys but was limited by variability. To address this, we applied decorrelation time (DT) mapping, a pixel-level technique that distinguishes intravascular (fast-moving) from extravascular (slow or stationary) NBs (44. This approach enables direct visualization of NB retention, a marker of increased vascular permeability. Intravascular NBs move rapidly with blood flow, whereas extravasated NBs remain largely immobile in interstitial spaces. Figure 1B shows representative log-scaled DT maps from sham and IRI kidneys across six 3.33-min acquisitions (~20 min total; full set in Supplementary Figure 3). Each map depicts the spatial distribution of DT during that interval. Sham kidneys showed uniform, short DT values consistent with rapid clearance, whereas IRI kidneys exhibited greater heterogeneity and progressively prolonged DT values, especially in later acquisitions 4-6.

To better capture these differences, we extracted histogram-based metrics from the DT maps, including mean DT (a global measure of NB retention over time), DT variance, and the number of pixels exceeding a 10-second DT threshold (defined from sham data), and compared these metrics between groups (Figure 1C-E). Here, DT variance reflects the spatial heterogeneity of pixel-wise DT values within individual kidneys (i.e., intra-animal variability), capturing patchy regions of prolonged NB retention rather than variability between animals or experimental groups. In the early phase (Aq 1-3; 0-10 min), mean DT, DT variance, and the number of pixels with DT > 10 s did not differ between IRI and sham kidneys (all p > 0.3). In the late phase (Aq 4-6; 10-20 min), IRI kidneys showed higher mean DT, significant at Aq 4 (p = 0.039; Glass's Δ ≈ 3.3) and trending at Aq 5-6. DT variance was likewise elevated, significant at Aq 4 (p = 0.028; Δ ≈ 25.3) and Aq 5 (p = 0.027; Δ ≈ 9.9), and higher but not significant at Aq 6. The number of pixels with DT > 10 s also increased in IRI kidneys, significant at Aq 4 and 5 (p = 0.025 and p = 0.050) and trending at Aq 6. These findings indicate greater late-phase NB persistence in IRI kidneys, with the lack of significance at Aq 6 likely due to reduced sample size and NB loss during prolonged insonation.

#### Parametric Maps

**Figure 2:**
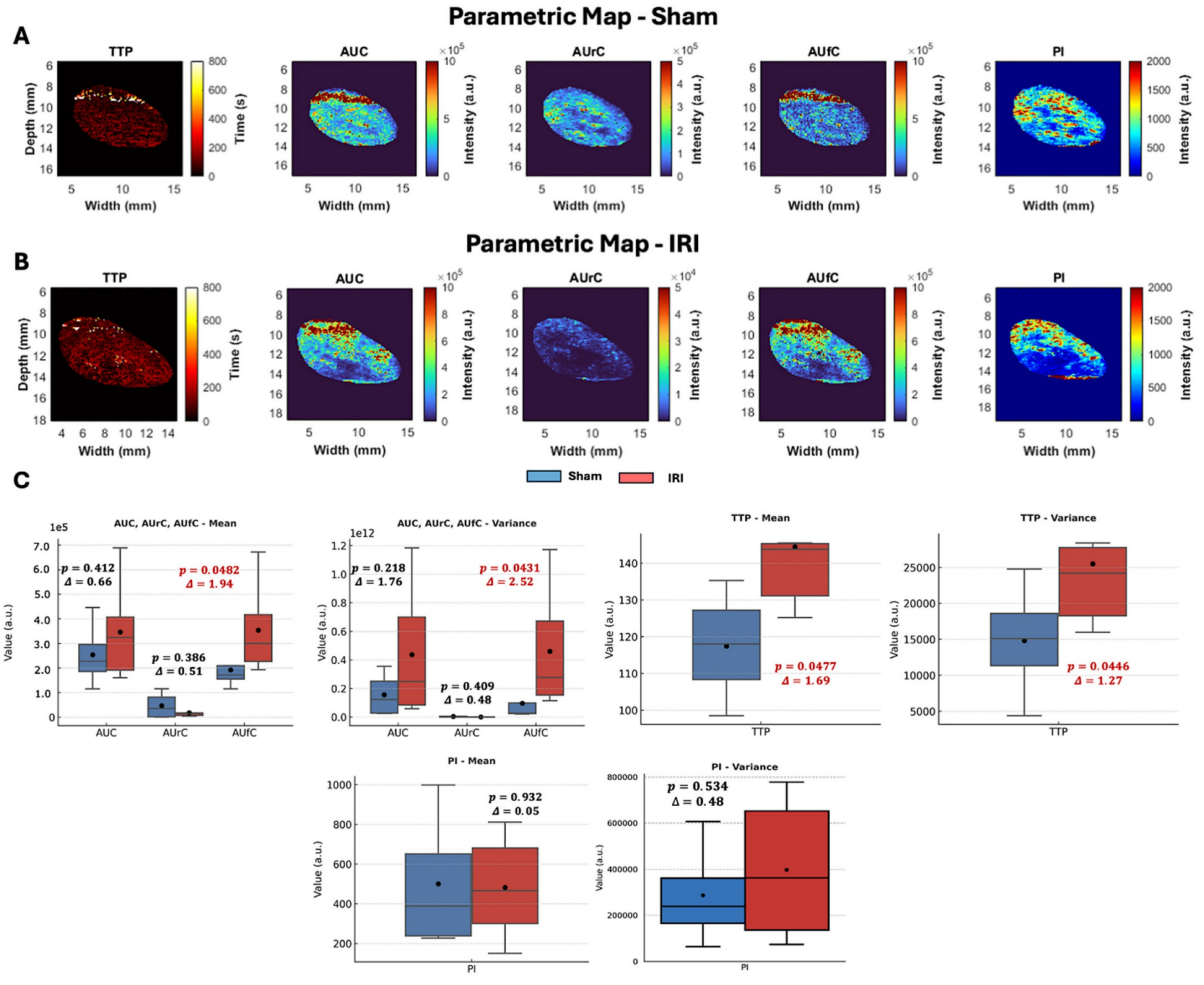
**CEUS parametric maps, pixel-value distributions, and group-level comparisons in sham and IRI kidneys.** (A-B) Representative parametric maps of TTP, AUC, AUrC, AUfC, and PI for (A) sham and (B) IRI kidneys. (C) Group-level comparison of kidney-averaged TIC parameters between sham and IRI animals. The mean and variance of each parameter were compared using Welch's t-test, and the effect size was calculated using Glass's Δ, which expresses the difference in means relative to the standard deviation of the control (Sham) group.

To further explore spatial differences in NB kinetics, we generated two-dimensional parametric maps of CEUS-derived TIC features (TTP, AUC, AUrC, AUfC, and PI) for sham and IRI kidneys (representative maps shown in Figure 2A-B, Supplementary Figure 4 shows the entire dataset).

Visual inspection revealed group differences despite some variability. Sham kidneys showed lower TTP values in the cortex and outer medulla, while IRI kidneys exhibited elevated TTP across larger regions, indicating delayed arrival. PI maps in shams were uniform, whereas IRI kidneys displayed heterogeneous enhancement with poorly perfused areas. AUC and AUrC were higher and more homogeneous in shams but patchier in IRI kidneys. Despite reduced inflow, AUfC was stronger in IRI kidneys, consistent with delayed washout and DT findings.

Histogram analyses of these parameters provided further insights Supplementary Figure 4B). We noted a leftward skewing of both PI and AUrC values in the IRI kidney, indicating lower PI and AUrC values throughout the IRI kidney that suggested impaired contrast inflow. In contrast, the AUfC demonstrated a rightward skewing in the IRI kidney, suggestive of delayed contrast washout.

To quantify these trends, kidney-averaged TIC parameters were compared between sham and IRI groups using t-tests, with effect sizes calculated as Glass's Δ (Figure 2C). While t-tests account for unequal variances and identify statistically significant group differences, they do not convey the magnitude of effect, which is critical in small-animal studies with limited power (46,47. Glass's Δ values ≥ 0.8 are considered large, consistent with biomedical thresholds for clinically meaningful differences 48.

In these analyses, TTP was significantly prolonged in IRI kidneys (p = 0.047, Glass's Δ = 1.69), with greater variability in its distribution (p = 0.044, Δ = 1.27) (Figure 2C), consistent with heterogeneous perfusion and disrupted vascular dynamics. AUfC values were also significantly elevated (p = 0.048, Δ = 1.94), with increased variability (p = 0.043, Δ = 2.52), indicating NB retention in IRI kidneys compared to shams. No significant group differences were observed for AUC (p = 0.412) or AUrC (p = 0.386), though AUrC was numerically lower in IRI kidneys.

Overall, TTP and AUfC were the most sensitive parameters, capturing delayed inflow and variable washout. Elevated AUfC further suggested NB extravasation, consistent with DT mapping findings (Figure 1B-E).

### Haralick Texture Feature Analysis

Haralick texture analysis was used on parametric maps to find spatial differences between sham and IRI kidneys. Features like contrast, energy, correlation, and homogeneity quantify image intensity patterns. Contrast shows local intensity variation, with higher values indicating more heterogeneity. Energy reflects uniformity; lower values mean a more disordered signal. Correlation gauges structural continuity, where lower values suggest disrupted flow. Homogeneity measures local smoothness, with lower values indicating abrupt changes. These methods have been used in US imaging to assess tissue structure heterogeneity 49-52.

**Figure 3:**
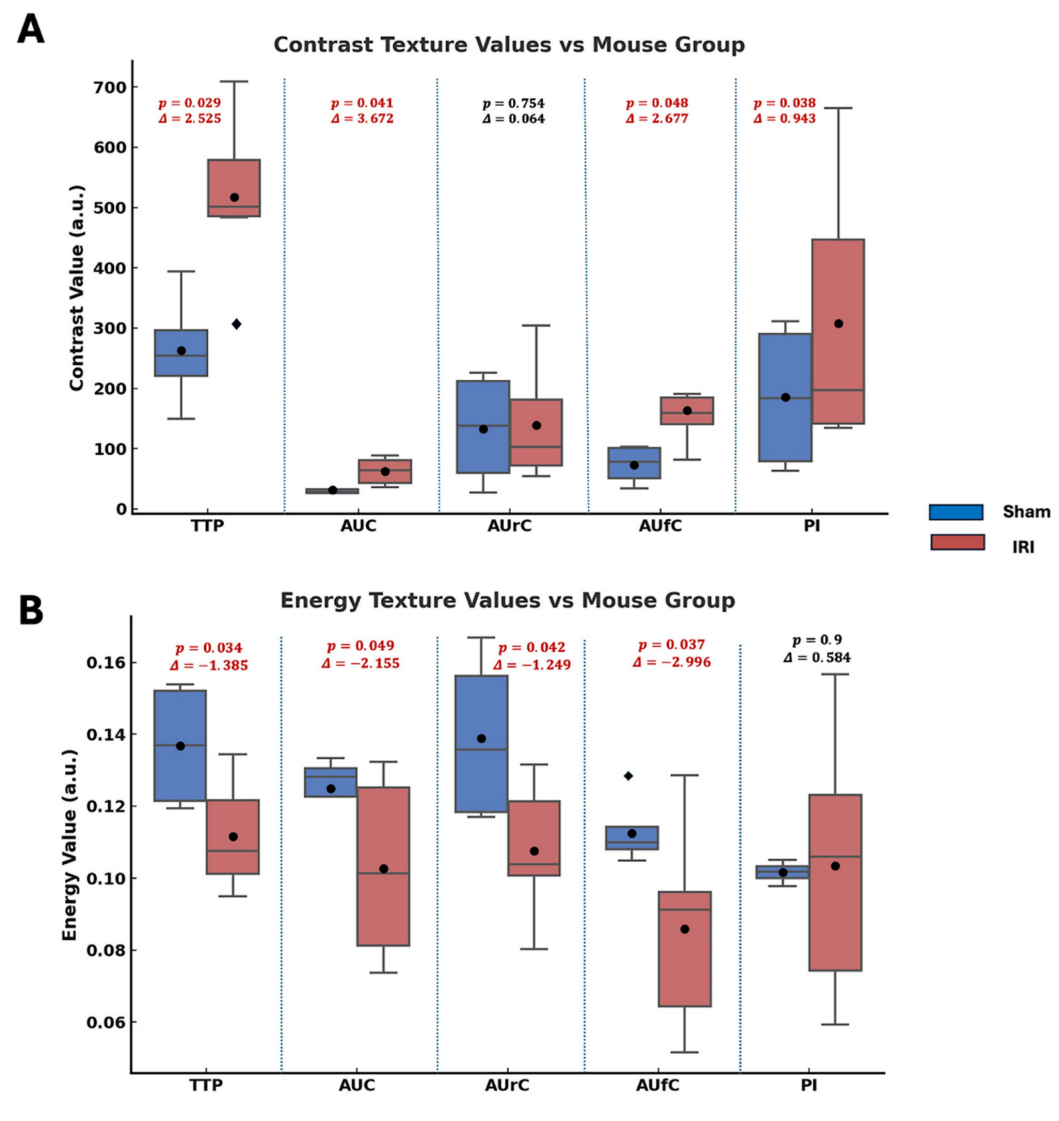

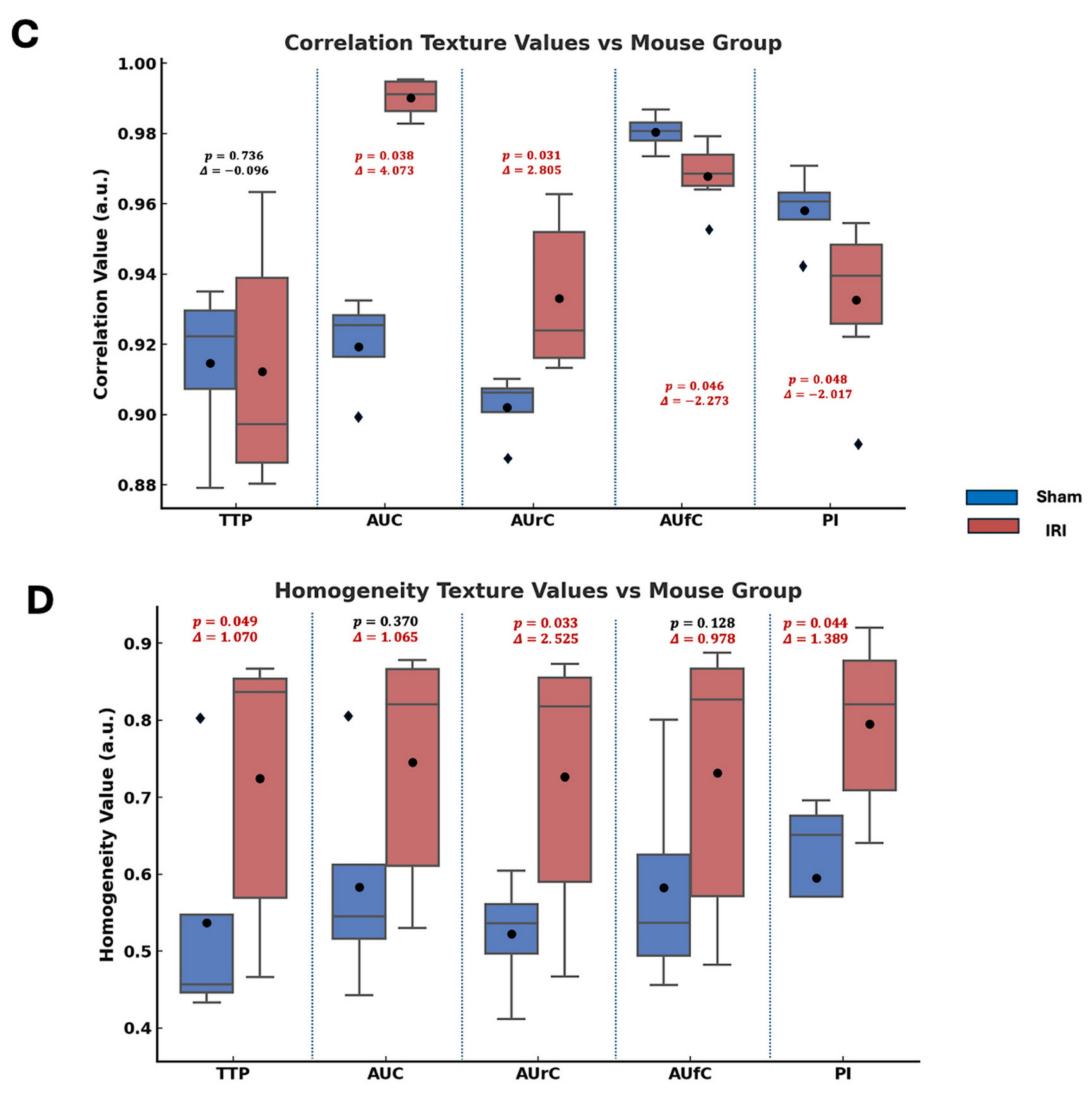
Statistical comparison of Haralick texture features between sham and IRI kidneys across TIC-derived parametric maps. Box plots showing differences in (A) contrast, (B) energy, (C) correlation, and (D) homogeneity texture values between sham (blue) and IRI (red) groups for each parametric map (TTP, AUC, AUrC, AUfC, and PI). Texture features were extracted from gray-level co-occurrence matrices (GLCMs) computed for each parametric map. Annotated above each pairwise comparison are the p-values from Welch's t-test and the Glass's delta (Δ) effect sizes (using sham group standard deviation).Across most parameters, IRI kidneys exhibited higher contrast and lower energy, indicating increased spatial heterogeneity and reduced textural regularity.

Figure 3 summarizes the statistical comparisons of these four key texture features between sham and IRI groups for each TIC-derived parameter map (TTP, AUC, AUrC, AUfC, PI). In TTP maps, the IRI group showed higher contrast and homogeneity, and lower energy. In AUC and AUfC maps, IRI kidneys also showed higher contrast and lower energy. Additionally, AUC maps showed higher correlation, while AUfC maps showed lower correlation.

Overall, the IRI group showed higher contrast (larger intensity differences between neighboring pixels) and lower energy (less uniform patterns), indicating greater spatial heterogeneity and disorder across several NB-CEUS parameters measured. Notably, PI energy did not differ significantly between groups.

#### Hematoxylin and Eosin (H&E) Staining and Inflammatory Cell Markers Quantifications

**Figure 4:**
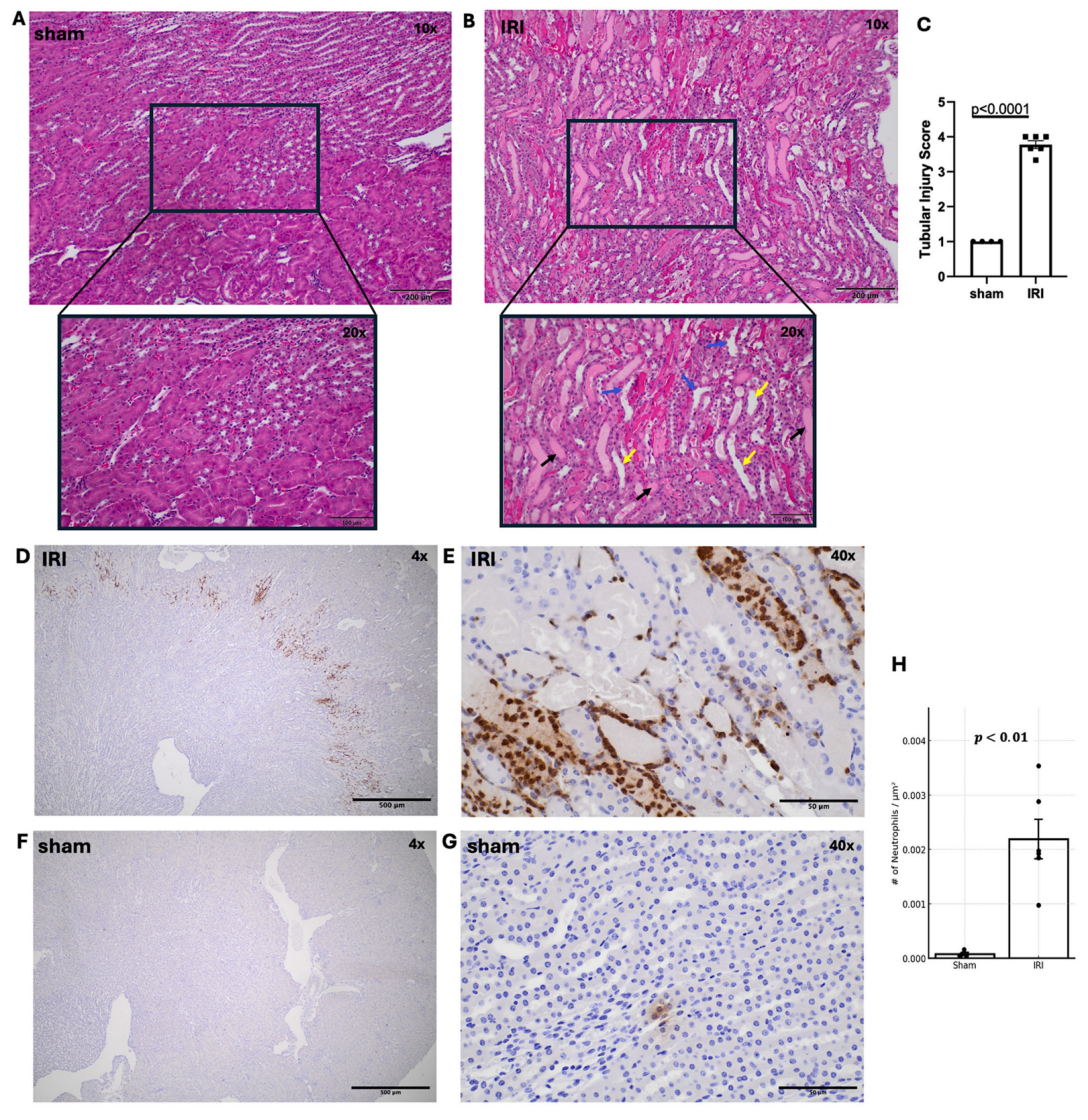
Histological assessment of tubular injury and neutrophil infiltration in sham and IRI kidneys. (A, B) Representative H&E-stained kidney sections from sham (A) and IRI (B) groups at 10X and 20X magnification. Tubular dilation (yellow arrows), cast formation (black arrows) and loss of brush border (blue arrows) are shown. (C) Quantification of tubular injury using a scoring system from 1 to 5, based on the extent of tubular damage in 2-3 fields of the outer medulla per kidney. p < 0.0001. (D-G) Immunohistochemical staining of IRI kidneys using anti-GR1 antibody to identify neutrophils. Neutrophil accumulation is seen throughout the outer medulla and perivascular regions at increasing magnifications (D: 4X, E: 40X. (F, G) Sham kidneys stained with anti-GR showing minimal neutrophil presence at both low (F: 4X) and higher (G: 40X) magnification. (H) Quantification of neutrophil infiltration reveals a significant increase in IRI kidneys compared to sham, as measured by the number of GR1+ neutrophils per µm². p < 0.01).

Following imaging, kidneys were collected for histology. Representative H&E-stained sham and IRI kidneys are shown in Figure 4A-B. Tubular injury in IRI kidneys was characterized by tubular dilation, cast formation, and loss of brush border, as illustrated in the high-magnification images. H&E revealed significantly higher tubular injury scores in IRI versus sham (Fig. 4A-C, p < 0.0001). Beyond tubular degeneration, IRI kidneys exhibited prominent neutrophil infiltration, particularly within the outer medulla and peritubular spaces (Figure 4D-E). These GR1⁺ neutrophils were frequently observed surrounding injured tubules and within areas of tubular necrosis with densities exceeding 0.002 cells/µm² in IRI kidneys versus <0.0001 cells/µm² in sham (Figure 4F-G). Consistent with our tubular injury findings, neutrophil distribution was also focal. Anti-F4/80 staining showed no signal in either group, indicating minimal macrophage recruitment 1-day post-IRI Supplementary Figure 5, consistent with reports that neutrophils dominate the early phase of renal IRI, while macrophages infiltrate later 35.

#### Immunofluorescent Confocal Microscopy

**Figure 5:**
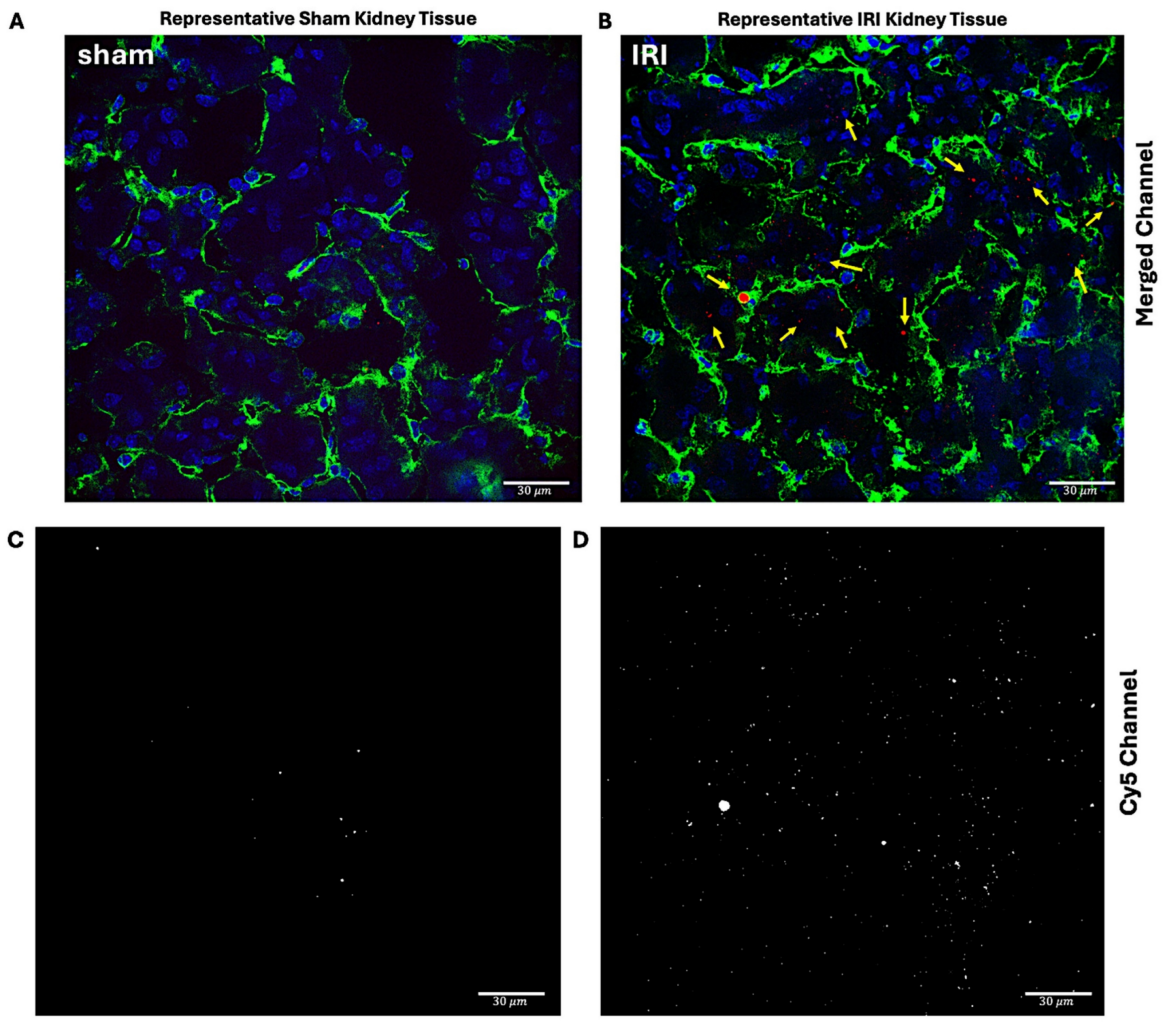

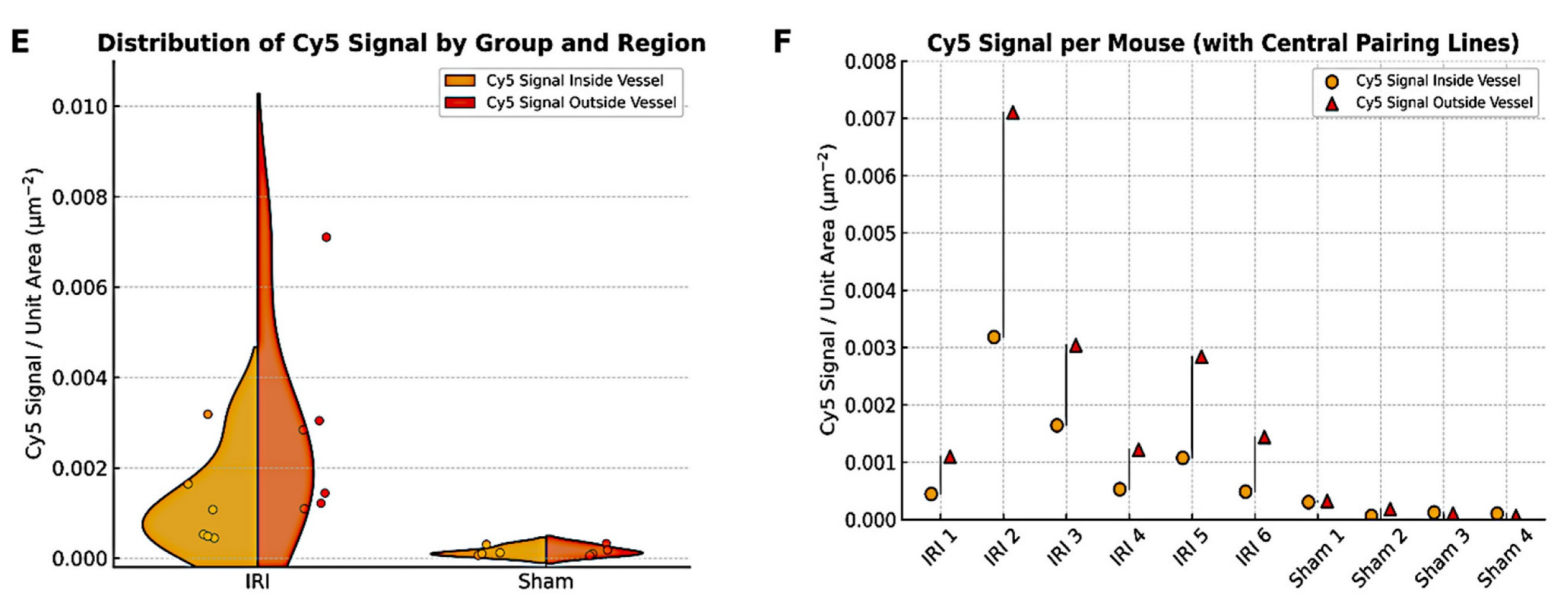
**Cy5-NB fluorescence signal distribution in sham and IRI kidney tissue.** Representative immunofluorescent imaging of sham (A, C) and IRI (B, D) kidneys to assess Cy5-tagged NB signal distribution. In the merged panels (A, B), nuclei (blue) are stained with DAPI, endothelial cells (green) with CD31, and Cy5-tagged NB signal (red, arrows) is highlighted. Panels (C, D) show the Cy5 channel, with the white color chosen to enhance contrast and represent the Cy5 signal in the tissue. The IRI kidney (B, D) exhibits an increased Cy5 signal compared to the sham kidney (A, C). Scale bars: 30 μm. Cy5 signal quantification inside and outside vessels in IRI and sham groups is shown in (E and F). Grouped data (E) showing Cy5 signal per unit area inside (yellow circle) and outside (red triangles) vessels. (F) Per mouse data, highlighting higher Cy5 signal levels in the IRI group compared to sham for both regions. Using ImageJ, vessel masks from the CD31 channel were applied to separate Cy5 signals inside and outside vessels, with analysis across 3-4 fields of view per mouse.

Immunofluorescent imaging was next performed to quantify the extent of NB extravasation in sham and IRI kidneys. To delineate the microvasculature, kidneys were stained with an antibody directed against CD31, a marker of endothelial cells (green). As shown in Figure 5A-D, intra- and extravascular NBs (red) were frequently observed in IRI kidneys, whereas only occasional NBs were noted in sham kidneys.

The total Cy5 signal per unit area was calculated for each field of view and plotted for IRI and sham groups across 3-4 fields. Figure 5E shows the average Cy5 distribution in each group, whereas Figure 5F summarizes data per mouse. IRI mice with greater extravascular NB signal also tended to have higher intravascular signal. To test robustness, the vessel threshold was varied by ±20% and reanalyzed, which did not change the conclusions Supplementary Figure 6.

#### Correlation between CEUS-derived Parameters and Histological Metrics

Since DT metrics showed the most pronounced differences between IRI and sham kidneys, we next correlated them with histologic measures of inflammation. Pearson correlation analyses were performed across six acquisitions using three biological indicators: 1 tubular injury scores from H&E sections, 2 extravasated Cy5-NB signal by fluorescence microscopy, and 3 neutrophil infiltration by GR1 immunostaining.

**Figure 6:**
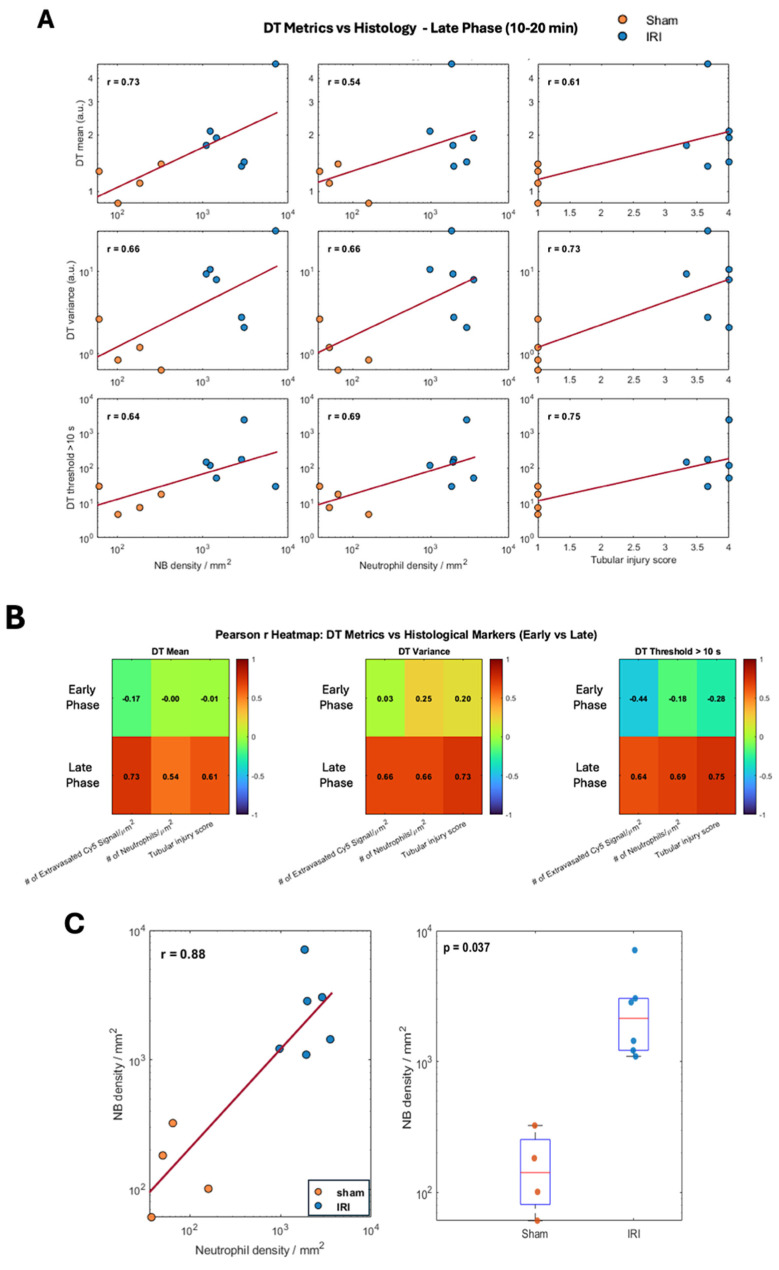
** Pearson Rank Correlation between CEUS imaging biomarkers and histological indicators of inflammation**. (A) Log-log Scatter plots showing Pearson rank (r) correlations between decorrelation time (DT) metrics, mean DT, DT variance, and number of pixels with DT >10 s with histological metrics (extravasated Cy5-labeled NBs signal/µm², neutrophil density (GR1⁺ cells/µm² and tubular injury score) during late phase (10-20 min). (B) Heatmaps summarize Pearson rank (r) values for DT metrics across early and late phases. (C) Left: Log-log scatterplot of extravasated nanobubble (NB) density versus neutrophil density across sham and IRI kidneys, with linear fit and Pearson correlation coefficient. Right: Comparison of extravasated NB density between sham and IRI kidneys; boxes indicate median and interquartile range, with individual animals shown; statistical comparison performed using Welch's two-sample t-test.

DT metrics (mean, variance, and number of pixels with DT >10 s) were correlated with histology using averaged values from early (Aq 1-3) and late (Aq 4-6) acquisitions. Associations are shown as log-log scatter plots in Figure 6A, with summary values in the heatmap (Figure 6B). Late-phase DT metrics correlated moderately to strongly with NB extravasation, neutrophil density, and tubular injury (r ≈ 0.54-0.75), whereas early-phase metrics showed weak or no correlations Supplementary Figure 7A). Extravascular NB density increased with neutrophil density across kidneys (Pearson r = 0.88), indicating a strong association with inflammatory cell infiltration. In addition, extravascular NB density was significantly higher in IRI kidneys compared with sham controls (Welch's t-test, p = 0.037).

Taken together, our data suggest that DT-based metrics, which quantify slow-moving NBs, capture not only NB extravasation but may also detect renal inflammation. Supplementary Figure 7B shows Pearson correlations between CEUS metrics and histology. AUfC correlated most strongly with extravasated NB signal (mean r ≈ 0.79; variance r ≈ 0.77), while correlations with neutrophil density were weaker (r ≈ 0.21-0.48). All CEUS metrics showed moderate associations with tubular injury (r ≈ 0.52-0.55). Overall, AUfC best tracked vascular leakage, whereas TTP showed only modest links.

## Discussion

Inflammation is a major consequence of ischemic AKI and a key driver of renal injury. At present, kidney inflammation can only be assessed by biopsy, which is invasive and prone to sampling bias. No reliable non-invasive method exists. Here, we demonstrate that NB-CEUS provides sensitive, real-time visualization of vascular and tissue changes in renal inflammation, independent of serum biomarkers or biopsy.

Conventional US only detects kidney size and echogenicity and cannot assess microvascular perfusion, inflammation, or tubular injury. Color Doppler measures renal blood flow and resistive indices but does not reliably reflect inflammation or microvascular damage (53. MB contrast agents are safe and echogenic but too large to cross endothelial gaps in inflamed vessels, restricting them to perfusion imaging. In contrast, NBs (100-500 nm) can extravasate through compromised vascular barriers and persist in inflamed tissue, enabling detection with standard US 28,38,54.

This study is the first to show that NBs detect both perfusion deficits and microvascular permeability in post-ischemic AKI. NB-CEUS resolves regional variations via pixel-level parametric maps, capturing heterogeneity in NB inflow and clearance missed by bulk metrics or biomarkers. IRI kidneys showed delayed TTP (inflow) and increased AUfC and DT (retention), indicating impaired flow and endothelial permeability. Parameter variance was also elevated, showing that both magnitude and spatial heterogeneity of CEUS metrics reflect microvascular injury (Figure 2).

The utility of NBs for assessing vascular permeability has also been demonstrated in other models. Notably, a study has shown that CEUS with submicron-sized NBs can detect pancreatic islet inflammation in type 1 diabetes 28, and extravasate into tumor interstitium in cancer models 55, supporting their broader translational applicability for monitoring vascular integrity across a range of disease states.

IRI induces endothelial swelling, detachment, and glycocalyx degradation, all contributing to vascular permeability and impaired barrier function 56,57. Although direct measurements of endothelial gap width in renal tissue are rare, several studies confirm that IRI disrupts the microvascular barrier via glycocalyx damage and increased permeability, allowing nanoscale agents such as NBs to extravasate 58-60. We leveraged this feature to test whether NB extravasation could serve as a non-invasive biomarker of post-IRI kidney inflammation. Histology showed NB retention in IRI but not sham kidneys (Figure 5), paralleled by delayed washout with increased AUfC (Figure 2) and prolonged DT (Figure 1). As microvascular permeability marks inflammation and injury, we quantified neutrophil accumulation and tubular injury, finding that DT correlated strongly with both (Figure 6).

Haralick texture analysis showed higher contrast and lower energy in IRI kidneys (Figure 3), consistent with greater spatial heterogeneity in perfusion and vascular dynamics. Similar findings have been reported in cancer and radiation injury studies 61,62. These changes likely reflect ischemia-induced microvascular disruption, where endothelial loss, leukocyte infiltration, and fibrosis generate heterogeneous perfusion. This aligns with known ischemic remodeling of renal tubules and vasculature, including pericyte detachment, capillary rarefaction, and interstitial fibrosis 63-65.

Although NB extravasation was observed in IRI kidneys, intravascular NB signal was also significantly higher (Figure 5). Prior studies show that IRI causes endothelial swelling/detachment, capillary narrowing, thrombosis, and leukocyte adhesion, collectively impairing flow and promoting vascular congestion 66. These structural alterations likely slowed NB clearance, leading to pooling or entrapment in obstructed capillaries. By contrast, sham kidneys showed minimal intravascular or interstitial NB retention, consistent with intact microvasculature and normal perfusion.

Variability in NB kinetics among mice may limit generalizability and reproducibility of kidney-specific outcomes. Natural biodistribution to the liver and spleen generates off-target signals, reducing renal specificity. Targeting NBs to adhesion molecules (e.g. VCAM-1) could improve specificity but raises challenges with stability, circulation, and scalability 38,67. Such targeting would alter DT mapping, as correlations would reflect endothelial binding rather than passive accumulation. While this may aid detection of subtle vascular damage or early inflammation, it also complicates interpretation and reproducibility. These trade-offs highlight both the promise and limitations of NB-based imaging. Optimizing NB size could further improve renal extravasation while minimizing off-target accumulation.

Inflammation after IRI is dynamic, with evolving vascular permeability and tissue remodeling. Serial NB-CEUS has the potential to define the temporal profile of NB inflow and retention, capturing the onset, peak, and resolution of inflammation while advancing mechanistic understanding of ischemic AKI. Because CEUS is already widely available in clinical practice, the incorporation of NBs could extend this modality from perfusion imaging to sensitive detection of microvascular permeability and inflammation. Our findings demonstrate that NB-CEUS yields quantitative, kidney-specific markers of injury that correlate with histology, positioning it as a promising non-invasive alternative to biopsy. Performed at the bedside, this approach could enable early diagnosis, therapeutic monitoring, and patient stratification. Key translational steps include validation of NB safety and stability in humans, but the workflow is directly compatible with existing US platforms, supporting rapid clinical adoption once regulatory approval is achieved.

## Conclusion

This study shows that CEUS with NBs enables non-invasive, quantitative imaging of kidney inflammation by capturing both vascular inflow and interstitial retention. Parameters such as TTP, AUfC, and DT metrics reflected impaired microvascular function and correlated with histologic evidence of injury and NB extravasation. A key advantage of this approach is its ability to provide kidney-specific and spatially resolved information, revealing heterogeneity in NB kinetics that global measurements obscure. This spatial insight reflects the patchy nature of inflammation and vascular damage after IRI. Haralick texture analysis further supported structural disruption, highlighting heterogeneity in perfusion patterns. Together, these findings establish CEUS with NBs as a promising tool for assessing renal inflammation, with potential for longitudinal monitoring and application across diverse kidney injury models.

## Supplementary Material

Supplementary figures and tables.

## References

[1] Chertow GM, Burdick E, Honour M, Bonventre JV, Bates DW (2005). Acute kidney injury, mortality, length of stay, and costs in hospitalized patients. J Am Soc Nephrol.

[2] Bonsib SM (2018). Urologic Diseases Germane to the Medical Renal Biopsy: Review of a Large Diagnostic Experience in the Context of the Renal Architecture and Its Environs. Adv Anat Pathol.

[3] Hull KL, Adenwalla SF, Topham P, Graham-Brown MP (2022). Indications and considerations for kidney biopsy: an overview of clinical considerations for the non-specialist. Clinical Medicine.

[4] Thurman JM, Gueler F (2018). Recent advances in renal imaging. F1000Res.

[5] Bouchard J, Malhotra R, Shah S, Kao YT, Vaida F, Gupta A (2015). Levels of Protein C and Soluble Thrombomodulin in Critically Ill Patients with Acute Kidney Injury: A Multicenter Prospective Observational Study. PLOS ONE.

[6] Parikh CR, Jani A, Mishra J, Ma Q, Kelly C, Barasch J (2006). Urine NGAL and IL-18 are predictive biomarkers for delayed graft function following kidney transplantation. Am J Transplant.

[7] Hellberg PO, Källskog O, Wolgast M (1990). Nephron function in the early phase of ischemic renal failure. Significance of erythrocyte trapping. Kidney Int.

[8] Wagener G, Jan M, Kim M, Mori K, Barasch JM, Sladen RN (2006). Association between increases in urinary neutrophil gelatinase-associated lipocalin and acute renal dysfunction after adult cardiac surgery. Anesthesiology.

[9] Esmon CT (2001). Protein C anticoagulant pathway and its role in controlling microvascular thrombosis and inflammation. Crit Care Med.

[10] Huang E, Mengel M, Clahsen-van Groningen MC, Jackson AM (2023). Diagnostic Potential of Minimally Invasive Biomarkers: A Biopsy-centered Viewpoint From the Banff Minimally Invasive Diagnostics Working Group. Transplantation.

[11] Mukherjee S, Bhaduri S, Harwood R, Murray P, Wilm B, Bearon R (2024). Multiparametric MRI based assessment of kidney injury in a mouse model of ischemia reperfusion injury. Sci Rep.

[12] Greite R, Derlin K, Hartung D, Chen R, Meier M, Gutberlet M (2021). Diffusion Weighted Imaging and T2 Mapping Detect Inflammatory Response in the Renal Tissue during Ischemia Induced Acute Kidney Injury in Different Mouse Strains and Predict Renal Outcome. Biomedicines.

[13] Li J, An C, Kang L, Mitch WE, Wang Y (2017). Recent Advances in Magnetic Resonance Imaging Assessment of Renal Fibrosis. Adv Chronic Kidney Dis.

[14] Muz B, Bandara N, Mpoy C, Sun J, Alhallak K, Azab F (2020). CXCR4-targeted PET imaging using 64Cu-AMD3100 for detection of Waldenström Macroglobulinemia. Cancer Biol Ther.

[15] Spatola L, Andrulli S (2016). Doppler ultrasound in kidney diseases: a key parameter in clinical long-term follow-up. J Ultrasound.

[16] Wei Q, Zhu Y, Zhen W, Zhang X, Shi Z, Zhang L (2022). Performance of resistive index and semi-quantitative power doppler ultrasound score in predicting acute kidney injury: A meta-analysis of prospective studies. PLOS ONE.

[17] Beloncle F, Rousseau N, Hamel JF, Donzeau A, Foucher AL, Custaud MA (2019). Determinants of Doppler-based renal resistive index in patients with septic shock: impact of hemodynamic parameters, acute kidney injury and predisposing factors. Ann Intensive Care.

[18] Wu SK, Chu PC, Chai WY, Kang ST, Tsai CH, Fan CH (2017). Characterization of Different Microbubbles in Assisting Focused Ultrasound-Induced Blood-Brain Barrier Opening. Sci Rep.

[19] Filippone A, Kirchin MA, Monteith J, Storto ML, Spinazzi A (2023). Safety of Lumason® (SonoVue®) in special populations and critically ill patients. Front Cardiovasc Med.

[20] Quaia E (2011). Assessment of tissue perfusion by contrast-enhanced ultrasound. Eur Radiol.

[21] Boesen EI, Crislip GR, Sullivan JC (2012). Use of ultrasound to assess renal reperfusion and P-selectin expression following unilateral renal ischemia. Am J Physiol Renal Physiol.

[22] Ren L, Zhao Y, Wang T, Tong Y, Zhao P, Nie F (2024). Ultrasound molecular imaging for early detection of acute renal ischemia-reperfusion injury. Bioeng Transl Med.

[23] Reinhart K, Bayer O, Brunkhorst F, Meisner M (2002). Markers of endothelial damage in organ dysfunction and sepsis. Crit Care Med.

[24] McDonald DM (1998). Endothelial Gaps: Plasma Leakage During Inflammation. Physiology.

[25] McDonald DM, Thurston G, Baluk P (1999). Endothelial gaps as sites for plasma leakage in inflammation. Microcirculation.

[26] McDonald DM (1994). Endothelial gaps and permeability of venules in rat tracheas exposed to inflammatory stimuli. Am J Physiol.

[27] Zhang J, Chen Y, Deng C, Zhang L, Sun Z, Wang J (2019). The Optimized Fabrication of a Novel Nanobubble for Tumor Imaging. Front Pharmacol.

[28] Ramirez DG, Abenojar E, Hernandez C, Lorberbaum DS, Papazian LA, Passman S (2020). Contrast-enhanced ultrasound with sub-micron sized contrast agents detects insulitis in mouse models of type1 diabetes. Nat Commun.

[29] Paefgen V, Doleschel D, Kiessling F (2015). Evolution of contrast agents for ultrasound imaging and ultrasound-mediated drug delivery. Front Pharmacol.

[30] Wu H, Abenojar EC, Perera R, Leon ACD, An T, Exner AA (2019). Time-intensity-curve Analysis and Tumor Extravasation of Nanobubble Ultrasound Contrast Agents. Ultrasound in Medicine and Biology.

[31] He X, Tolosa MF, Zhang T, Goru SK, Ulloa Severino L, Misra PS Myofibroblast YAP/TAZ activation is a key step in organ fibrogenesis. JCI Insight. 7(4):e146243.

[32] Leon A de, Perera R, Hernandez C, Cooley M, Jung O, Jeganathan S (2019). Contrast enhanced ultrasound imaging by nature-inspired ultrastable echogenic nanobubbles. Nanoscale.

[33] Abenojar EC, Nittayacharn P, de Leon AC, Perera R, Wang Y, Bederman I (2019). Effect of Bubble Concentration on the in Vitro and in Vivo Performance of Highly Stable Lipid Shell-Stabilized Micro- and Nanoscale Ultrasound Contrast Agents. Langmuir.

[34] Hernandez C, Abenojar EC, Hadley J, de Leon AC, Coyne R, Perera R (2019). Sink or float?. Characterization of shell-stabilized bulk nanobubbles using a resonant mass measurement technique †Electronic supplementary information (ESI) available: Experimental details, supporting information. See DOI: 10.1039/c8nr08763f. Nanoscale.

[35] Holderied A, Kraft F, Marschner JA, Weidenbusch M, Anders HJ (2020). “Point of no return” in unilateral renal ischemia reperfusion injury in mice. Journal of Biomedical Science.

[36] Stroo I, Stokman G, Teske GJD, Raven A, Butter LM, Florquin S (2010). Chemokine expression in renal ischemia/reperfusion injury is most profound during the reparative phase. International Immunology.

[37] Wang L, Vijayan V, Jang MS, Thorenz A, Greite R, Rong S (2019). Labile Heme Aggravates Renal Inflammation and Complement Activation After Ischemia Reperfusion Injury. Front Immunol [Internet]. 2019 Dec 20 [cited 2025 May 14];10. Available from: https://www.frontiersin.org/journals/immunology/articles/10.3389/fimmu.

[38] Wegierak D, Nittayacharn P, Cooley MB, Berg FM, Kosmides T, Durig D (2024). Nanobubble Contrast Enhanced Ultrasound Imaging: A Review. Wiley Interdiscip Rev Nanomed Nanobiotechnol.

[39] Sheeran PS, Daghighi Y, Yoo K, Williams R, Cherin E, Foster FS (2016). Image-Guided Ultrasound Characterization of Volatile Sub-Micron Phase-Shift Droplets in the 20-40 MHz Frequency Range. Ultrasound Med Biol.

[40] Pabla N, Scindia Y, Gigliotti J, Bajwa A, Pabla N, Scindia Y (2025). Mouse Models of Acute Kidney Injury. In: Preclinical Animal Modeling in Medicine [Internet]. IntechOpen; 2021 [cited.

[41] Turco S, Wijkstra H, Mischi M (2016). Mathematical Models of Contrast Transport Kinetics for Cancer Diagnostic Imaging: A Review. IEEE Rev Biomed Eng.

[42] Haralick RM, Shanmugam K, Dinstein I (1973). Textural Features for Image Classification. IEEE Transactions on Systems, Man, and Cybernetics.

[43] Rolland Y, Bézy-Wendling J, Gestin H, Bruno A, Duvauferrier R, Morcet N (1995). [Analysis of texture in medical imaging. Review of the literature]. Ann Radiol (Paris).

[44] Wegierak D, Cooley MB, Perera R, Wulftange WJ, Gurkan UA, Kolios MC (2024). Decorrelation Time Mapping as an Analysis Tool for Nanobubble-Based Contrast Enhanced Ultrasound Imaging. IEEE Trans Med Imaging.

[45] Yamamoto T, Tada T, Brodsky SV, Tanaka H, Noiri E, Kajiya F (2002). Intravital videomicroscopy of peritubular capillaries in renal ischemia. American Journal of Physiology-Renal Physiology.

[46] Lakens D (2013). Calculating and reporting effect sizes to facilitate cumulative science: a practical primer for t-tests and ANOVAs. Front Psychol [Internet]. 2013 Nov 26 [cited 2025 May 19];4. Available from: https://www.frontiersin.org/journals/psychology/articles/10.3389/fpsyg.

[47] Primary Secondary, Meta-Analysis of Research1 - GENE V GLASS 1976 [Internet] [cited 2025 May 19]. Available from: https://journals.sagepub.com/doi/abs/10.3102/0013189x005010003.

[48] Johnson HR, Gunder LC, Gillette A, Sleiman H, Rademacher BL, Meske LM (2024). Preclinical Models of Anal Cancer Combined-Modality Therapy. Journal of Surgical Research.

[49] Cao G tao, Shi P fei, Hu B (2005). Liver fibrosis identification based on ultrasound images captured under varied imaging protocols. J Zhejiang Univ Sci B.

[50] Sadeghi-Naini A, Suraweera H, Tran WT, Hadizad F, Bruni G, Rastegar RF (2017). Breast-Lesion Characterization using Textural Features of Quantitative Ultrasound Parametric Maps. Sci Rep.

[51] Gao S, Peng Y, Guo H, Liu W, Gao T, Xu Y (2014). Texture analysis and classification of ultrasound liver images. Bio-Medical Materials and Engineering.

[52] Al-Hasani M, Sultan LR, Sagreiya H, Cary TW, Karmacharya MB, Sehgal CM (2022). Ultrasound Radiomics for the Detection of Early-Stage Liver Fibrosis. Diagnostics (Basel).

[53] Moriconi D, Mengozzi A, Duranti E, Cappelli F, Taddei S, Nannipieri M (2023). The renal resistive index is associated with microvascular remodeling in patients with severe obesity. Journal of Hypertension.

[54] Zhao X, Pellow C, Goertz DE (2023). Intravital imaging and cavitation monitoring of antivascular ultrasound in tumor microvasculature. Theranostics.

[55] Pellow C, Abenojar EC, Exner AA, Zheng G, Goertz DE (2020). Concurrent visual and acoustic tracking of passive and active delivery of nanobubbles to tumors. Theranostics.

[56] Sutton TA, Fisher CJ, Molitoris BA (2002). Microvascular endothelial injury and dysfunction during ischemic acute renal failure. Kidney Int.

[57] Guan Z, Gobé G, Willgoss D, Endre ZH (2006). Renal endothelial dysfunction and impaired autoregulation after ischemia-reperfusion injury result from excess nitric oxide. Am J Physiol Renal Physiol.

[58] Kumar S, Molitoris BA (2015). Renal Endothelial Injury and Microvascular Dysfunction in Acute Kidney Injury. Semin Nephrol.

[59] Duni A, Liakopoulos V, Koutlas V, Pappas C, Mitsis M, Dounousi E (2021). The Endothelial Glycocalyx as a Target of Ischemia and Reperfusion Injury in Kidney Transplantation—Where Have We Gone So Far?. International Journal of Molecular Sciences.

[60] Tietjen GT, Hosgood SA, DiRito J, Cui J, Deep D, Song E (2017). Nanoparticle targeting to the endothelium during normothermic machine perfusion of human kidneys. Sci Transl Med.

[61] Vrbik I, Nest SJV, Meksiarun P, Loeppky J, Brolo A, Lum JJ (2019). Haralick texture feature analysis for quantifying radiation response heterogeneity in murine models observed using Raman spectroscopic mapping. PLOS ONE.

[62] Caruso D, Zerunian M, Ciolina M, de Santis D, Rengo M, Soomro MH (2018). Haralick's texture features for the prediction of response to therapy in colorectal cancer: a preliminary study. Radiol Med.

[63] Kramann R, Humphreys BD (2014). Kidney Pericytes: Roles in Regeneration and Fibrosis. Seminars in Nephrology.

[64] Chou YH, Pan SY, Shih HM, Lin SL (2023). Update of pericytes function and their roles in kidney diseases. J Formos Med Assoc.

[65] Duffield JS (2014). Cellular and molecular mechanisms in kidney fibrosis. J Clin Invest.

[66] Sharfuddin AA, Molitoris BA (2011). Pathophysiology of ischemic acute kidney injury. Nat Rev Nephrol.

[67] Chen X, Xu L, Chen C, Huang Q, Hu J (2025). Recent advances in ultrasound-targeted nanobubbles combined with cancer immunotherapy: Mechanisms, applications, and challenges. Fundamental Research [Internet]. 2024 Nov 13 [cited.

