# Supplementary material for: Quantification of Kidney Inflammation Using Nanobubble-mediated Contrast Enhanced Ultrasound
Source: Nanotheranostics. 2026 Feb 11;10:38–53. doi: 10.7150/ntno.126443 (PMC12923311; doi:10.7150/ntno.126443)
Supplement: Supplementary file 1 — Supplementary figures and tables. [file ntnov10p0038s1.pdf]

IX. Supplementary Figures

Table of Contents

1. **Supplementary Figure 1:** NB injection and CEUS imaging setup…………………. 24
2. **Supplementary Figure 2:** CEUS imaging TIC parameters following Cy5 NB injection in Sham and IRI kidney…………………………………………….. 24
3. **Supplementary Figure 3:** Spatial and statistical assessment of decorrelation time maps in IRI and sham kidneys……………….…………………………………………… 25-27
4. **Supplementary Figure 4:** Parametric mapping and histogram analysis of CEUS-derived metrics in individual kidneys……………………………………………………. 28-30
5. **Supplementary Figure 5:** F4/80 immunohistochemical staining reveals no macrophage infiltration in both Sham and IRI kidneys ………………………………………… 31
6. **Supplementary Figure 6:** Robustness of Cy5 signal distribution analysis to vessel mask variation………………………………………………………………………… 32
7. **Supplementary Figure 7:** Early phase Pearson Rank Correlations Between Imaging Metrics and Histological Markers of Kidney Inflammation…………………………. 33

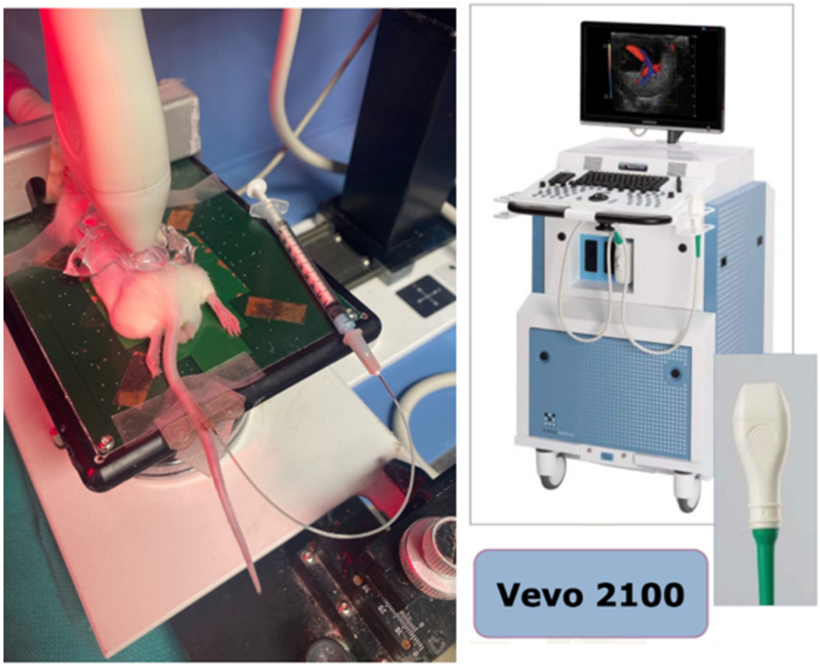

Supplementary Figure 1: NB injection and CEUS imaging setup: *A mouse under anesthesia is positioned on a heated platform for CEUS imaging. NBs were administered through a tail vein injection using a butterfly catheter. (B) Illustration of the Vevo 2100 imaging system equipped with the MS250 transducer.*

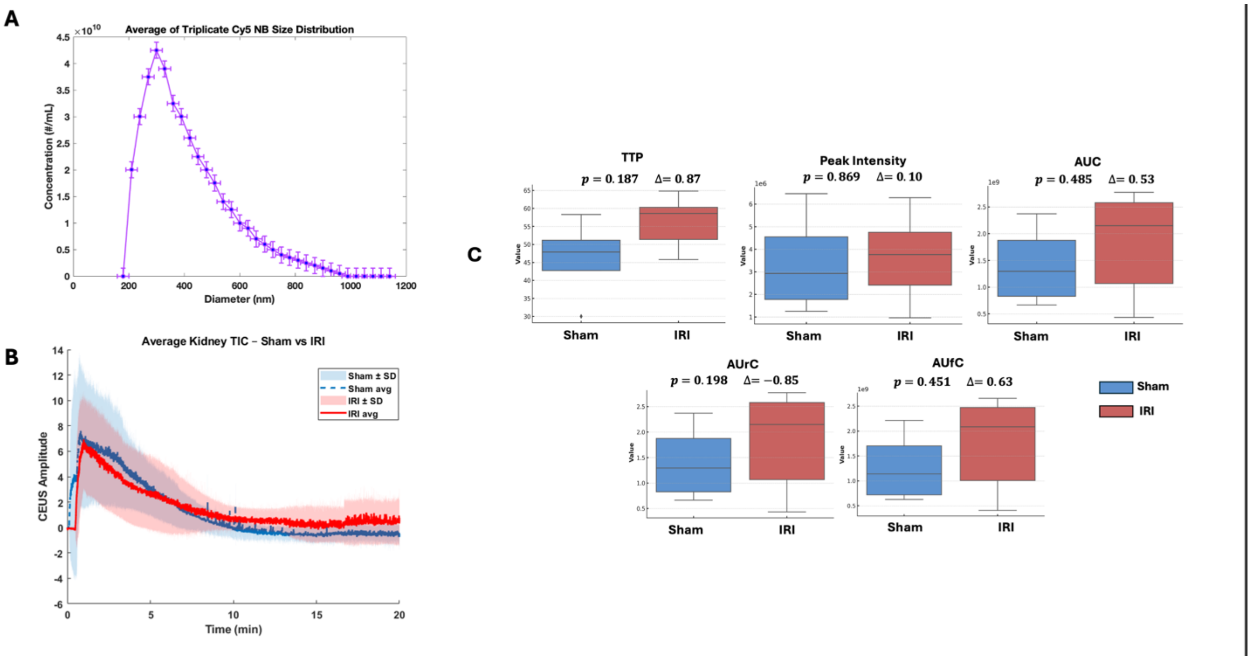

Supplementary Figure 2: CEUS imaging TIC parameters following Cy5-NB injection in Sham and IRI kidneys. *Representative CEUS images and TICs of kidney perfusion in sham and IRI groups following Cy5-NB injection. (A) Size distribution of Cy5 NBs with an average diameter of* $348 \pm 21\ nm$ *and a total concentration of* $3.99\ \pm 0.12 \times 10^{11}\ NB/mL$ *measured with the Archimedes Metrology system.(B) Group-averaged TICs (mean ± SD). (C) ROI-averaged quantification of TIC parameters across all mice for each group. Error bars represent standard deviation.*

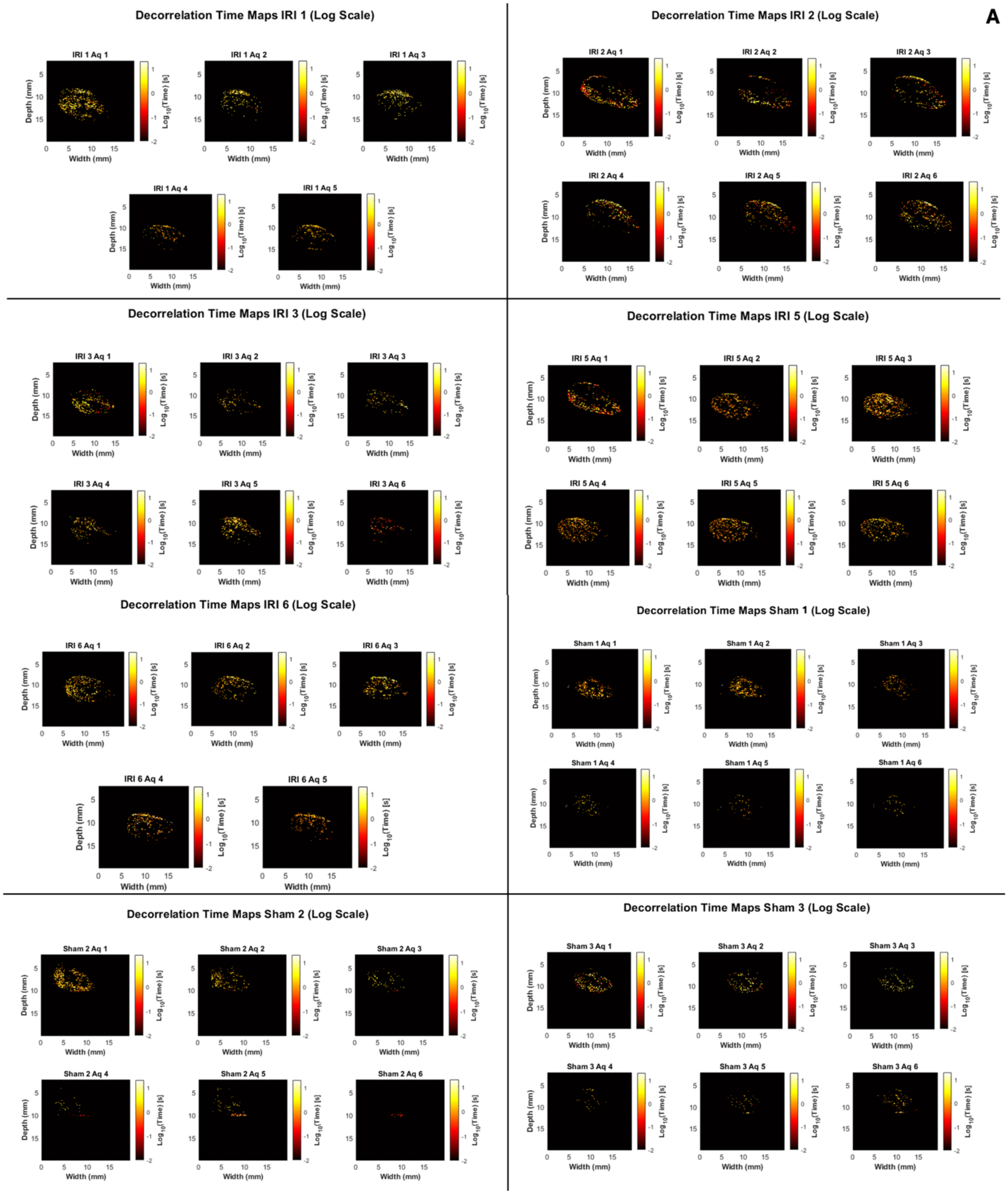
A
Decorrelation Time Maps IRI 1 (Log Scale)
IRI 1 Aq 1
IRI 1 Aq 2
IRI 1 Aq 3
IRI 1 Aq 4
IRI 1 Aq 5
Decorrelation Time Maps IRI 2 (Log Scale)
IRI 2 Aq 1
IRI 2 Aq 2
IRI 2 Aq 3
IRI 2 Aq 4
IRI 2 Aq 5
IRI 2 Aq 6
Decorrelation Time Maps IRI 3 (Log Scale)
IRI 3 Aq 1
IRI 3 Aq 2
IRI 3 Aq 3
IRI 3 Aq 4
IRI 3 Aq 5
IRI 3 Aq 6
Decorrelation Time Maps IRI 5 (Log Scale)
IRI 5 Aq 1
IRI 5 Aq 2
IRI 5 Aq 3
IRI 5 Aq 4
IRI 5 Aq 5
IRI 5 Aq 6
Decorrelation Time Maps IRI 6 (Log Scale)
IRI 6 Aq 1
IRI 6 Aq 2
IRI 6 Aq 3
IRI 6 Aq 4
IRI 6 Aq 5
Decorrelation Time Maps Sham 1 (Log Scale)
Sham 1 Aq 1
Sham 1 Aq 2
Sham 1 Aq 3
Sham 1 Aq 4
Sham 1 Aq 5
Sham 1 Aq 6
Decorrelation Time Maps Sham 2 (Log Scale)
Sham 2 Aq 1
Sham 2 Aq 2
Sham 2 Aq 3
Sham 2 Aq 4
Sham 2 Aq 5
Sham 2 Aq 6
Decorrelation Time Maps Sham 3 (Log Scale)
Sham 3 Aq 1
Sham 3 Aq 2
Sham 3 Aq 3
Sham 3 Aq 4
Sham 3 Aq 5
Sham 3 Aq 6
Depth (mm)
Width (mm)
Log_{10}(Time) [s]

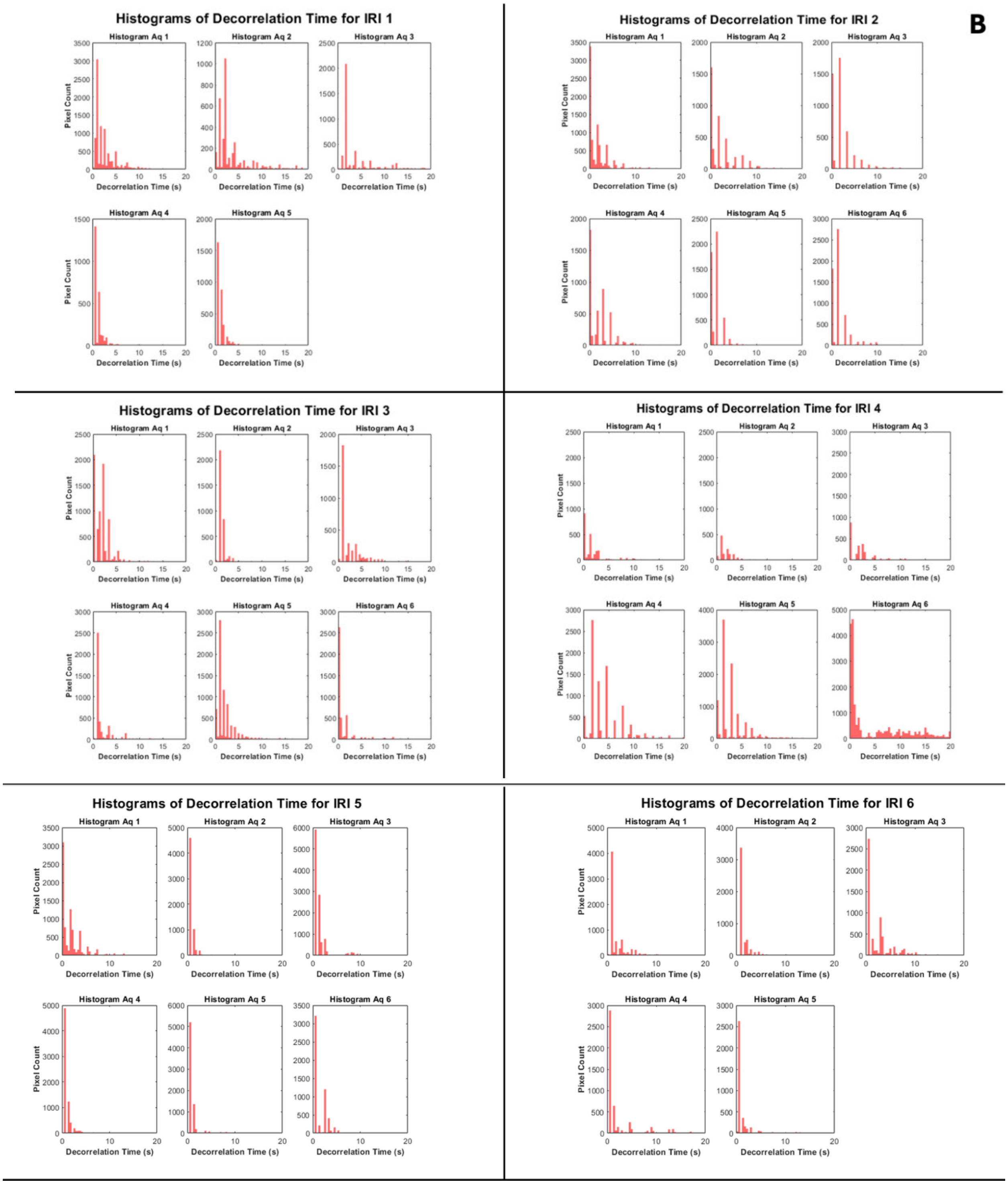
B
Histograms of Decorrelation Time for IRI 1
Histogram Aq 1
Histogram Aq 2
Histogram Aq 3
Histogram Aq 4
Histogram Aq 5
Histograms of Decorrelation Time for IRI 2
Histogram Aq 1
Histogram Aq 2
Histogram Aq 3
Histogram Aq 4
Histogram Aq 5
Histogram Aq 6
Histograms of Decorrelation Time for IRI 3
Histogram Aq 1
Histogram Aq 2
Histogram Aq 3
Histogram Aq 4
Histogram Aq 5
Histogram Aq 6
Histograms of Decorrelation Time for IRI 4
Histogram Aq 1
Histogram Aq 2
Histogram Aq 3
Histogram Aq 4
Histogram Aq 5
Histogram Aq 6
Histograms of Decorrelation Time for IRI 5
Histogram Aq 1
Histogram Aq 2
Histogram Aq 3
Histogram Aq 4
Histogram Aq 5
Histogram Aq 6
Histograms of Decorrelation Time for IRI 6
Histogram Aq 1
Histogram Aq 2
Histogram Aq 3
Histogram Aq 4
Histogram Aq 5
Pixel Count
Decorrelation Time (s)

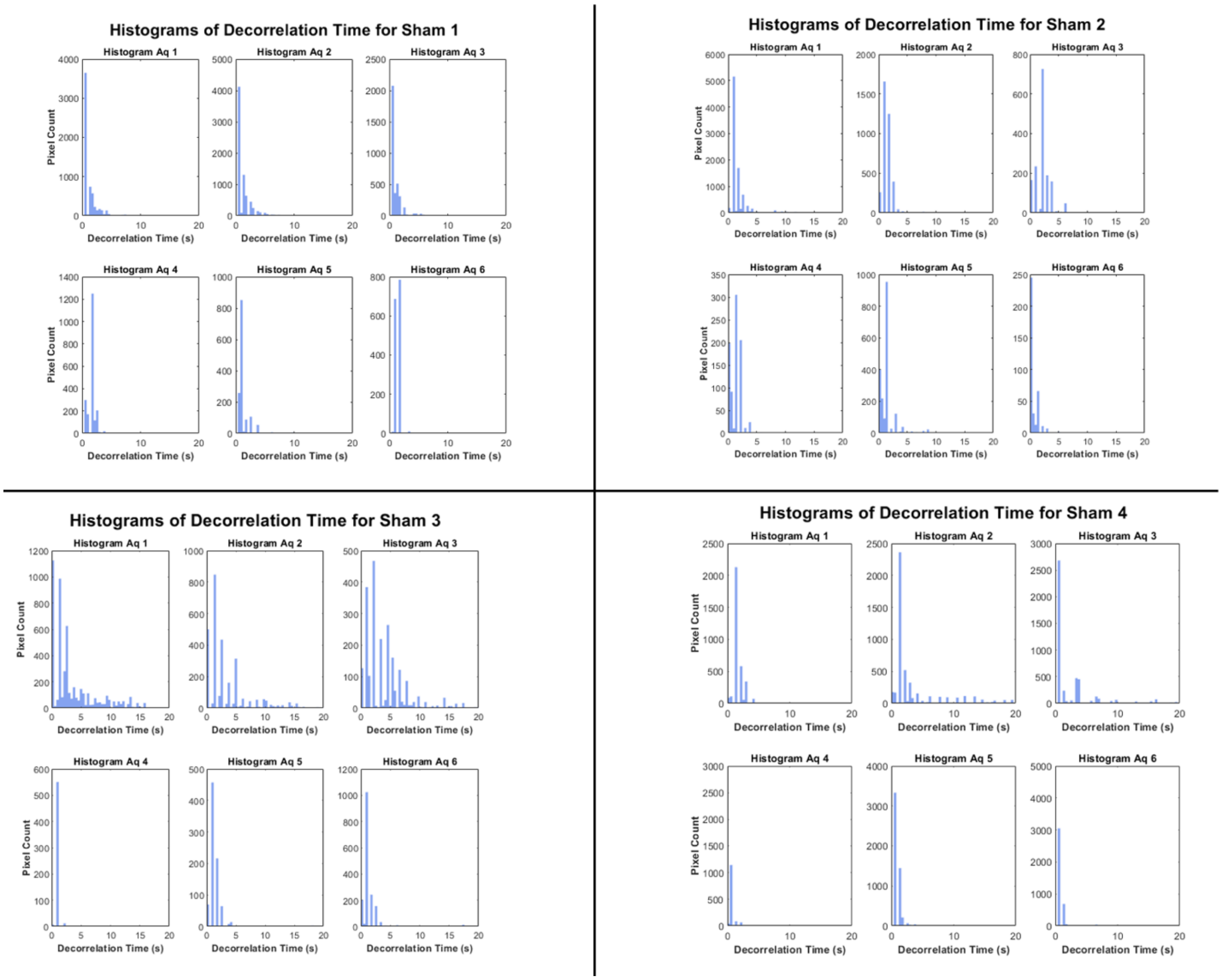

***Supplementary Figure 3: Spatial and statistical assessment of decorrelation time maps in IRI and sham kidneys**. **(A)** Log-scale decorrelation time (DT) maps were generated for each remaining animal (i.e. excluding those presented in Figure 2) in the IRI (n=5) and sham (n=3) groups across six acquisitions (Ag 1–6). These maps visualize the spatial distribution of nonlinear contrast agent motion, with longer DTs reflecting reduced particle mobility typically associated with extravasation or interstitial retention. **(B)** Corresponding histograms display the pixel-wise distribution of decorrelation times for each acquisition for IRI (n=6) and sham (n=4). IRI kidneys consistently show broader distributions and longer DT tails, indicative of altered contrast kinetics and increased vascular permeability post-injury. Sham kidneys exhibit more compact distributions with faster DTs, reflecting primarily intravascular contrast behavior.*

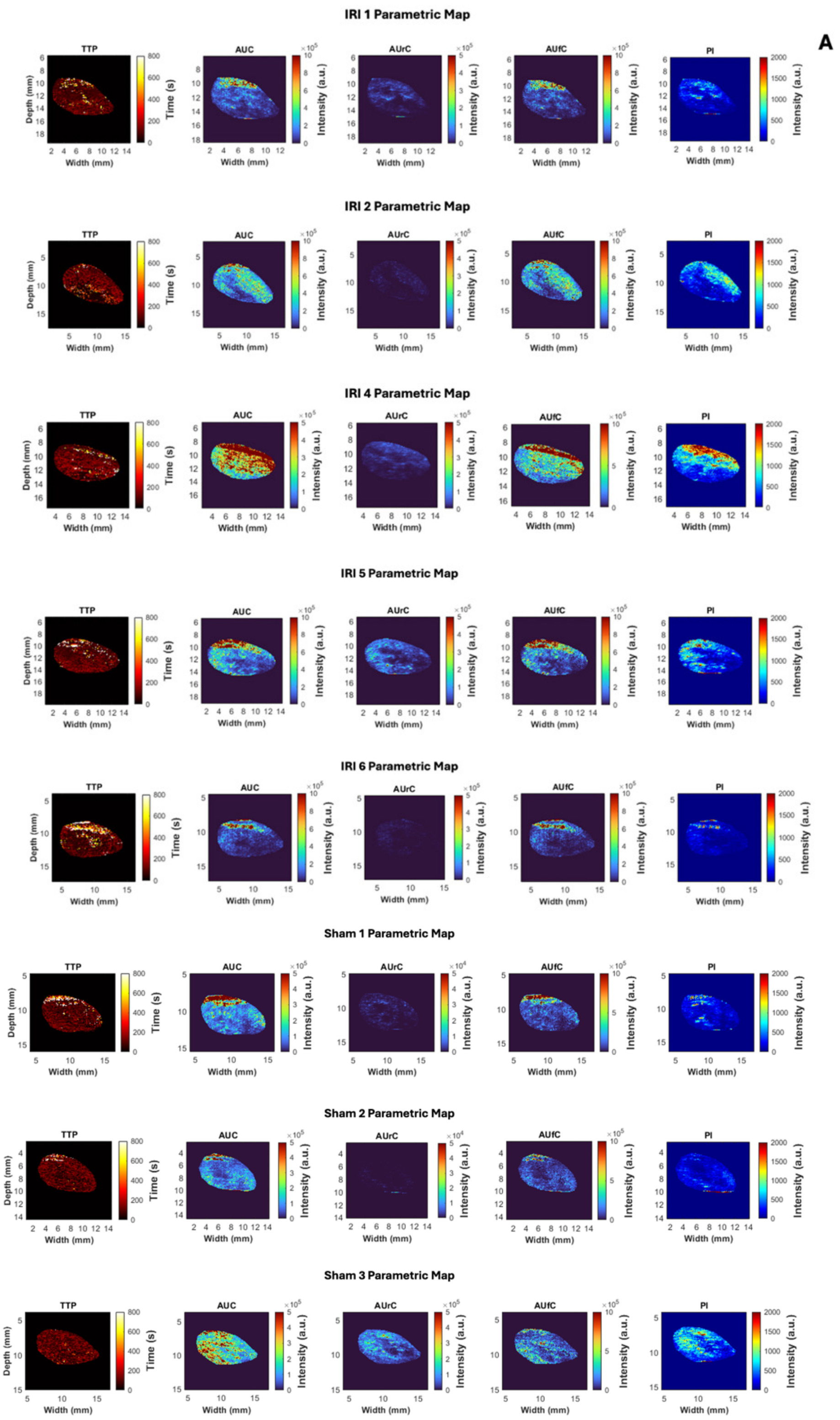
A
IRI 1 Parametric Map
IRI 2 Parametric Map
IRI 4 Parametric Map
IRI 5 Parametric Map
IRI 6 Parametric Map
Sham 1 Parametric Map
Sham 2 Parametric Map
Sham 3 Parametric Map
TTP
AUC
AUrC
AUfC
PI
Depth (mm)
Width (mm)
Time (s)
Intensity (a.u.)

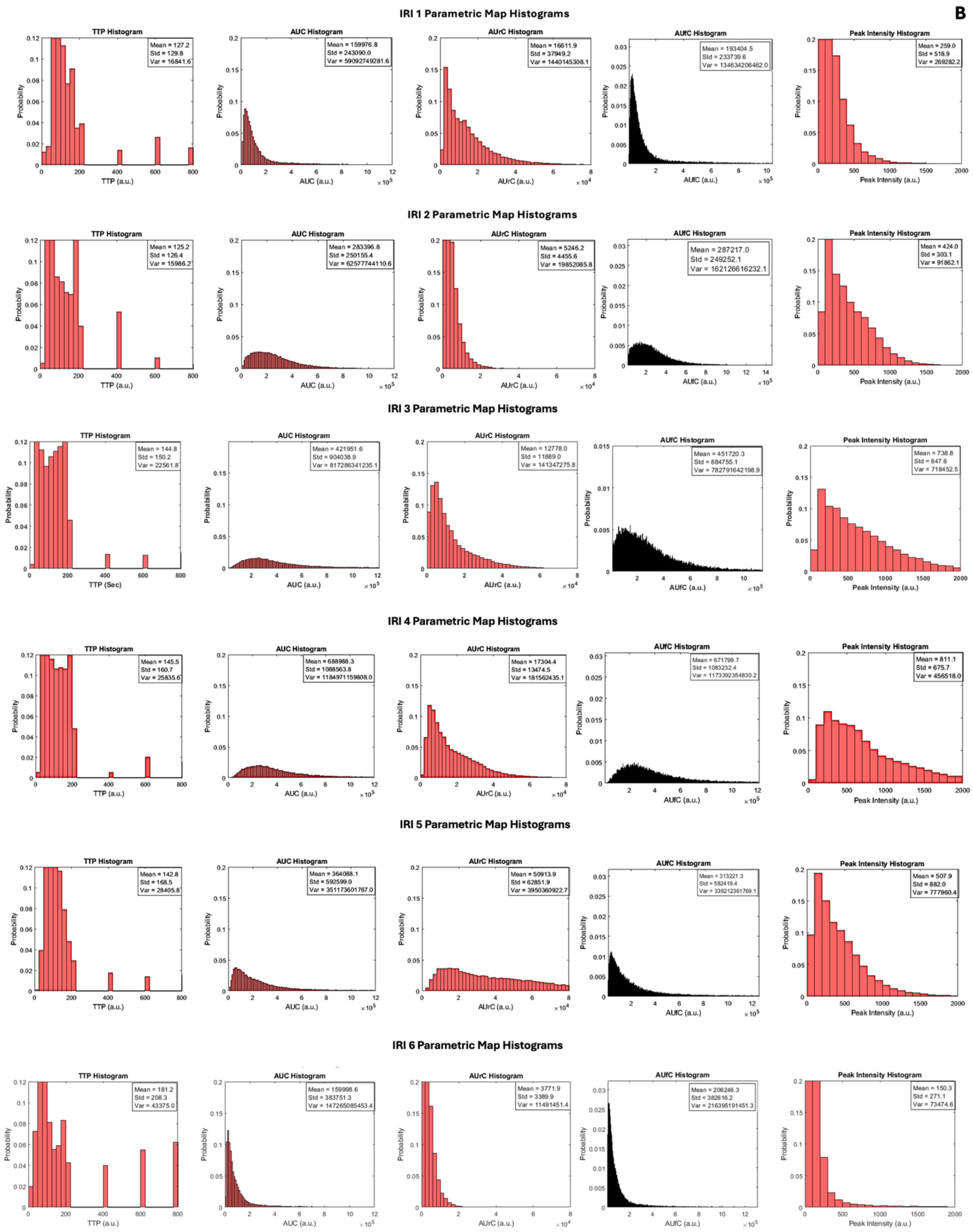
B
IRI 1 Parametric Map Histograms
TTP Histogram
Mean = 127.2
Std = 129.8
Var = 16841.6
AUC Histogram
Mean = 159976.8
Std = 243090.0
Var = 59092749281.6
AUrC Histogram
Mean = 16611.9
Std = 37949.2
Var = 1440145308.1
AUfC Histogram
Mean = 193404.5
Std = 233739.6
Var = 134634206462.0
Peak Intensity Histogram
Mean = 259.0
Std = 518.9
Var = 269282.2
IRI 2 Parametric Map Histograms
TTP Histogram
Mean = 125.2
Std = 126.4
Var = 15986.2
AUC Histogram
Mean = 283396.8
Std = 250155.4
Var = 62577744110.6
AUrC Histogram
Mean = 5246.2
Std = 4455.6
Var = 19852085.8
AUfC Histogram
Mean = 287217.0
Std = 249252.1
Var = 162126616232.1
Peak Intensity Histogram
Mean = 424.0
Std = 303.1
Var = 91862.1
IRI 3 Parametric Map Histograms
TTP Histogram
Mean = 144.8
Std = 150.2
Var = 22561.8
AUC Histogram
Mean = 421951.6
Std = 904038.9
Var = 817286341235.1
AUrC Histogram
Mean = 12778.0
Std = 11889.0
Var = 141347275.8
AUfC Histogram
Mean = 451720.3
Std = 884755.1
Var = 782791642198.9
Peak Intensity Histogram
Mean = 738.8
Std = 847.6
Var = 718452.5
IRI 4 Parametric Map Histograms
TTP Histogram
Mean = 145.5
Std = 160.7
Var = 25835.6
AUC Histogram
Mean = 688988.3
Std = 1088563.8
Var = 1184971159808.0
AUrC Histogram
Mean = 17304.4
Std = 13474.5
Var = 181562435.1
AUfC Histogram
Mean = 671799.7
Std = 1083232.4
Var = 1173392354830.2
Peak Intensity Histogram
Mean = 811.1
Std = 675.7
Var = 456518.0
IRI 5 Parametric Map Histograms
TTP Histogram
Mean = 142.8
Std = 168.5
Var = 28405.8
AUC Histogram
Mean = 364088.1
Std = 592599.0
Var = 351173601767.0
AUrC Histogram
Mean = 50913.9
Std = 62851.9
Var = 3950360922.7
AUfC Histogram
Mean = 313221.3
Std = 582419.4
Var = 339212361769.1
Peak Intensity Histogram
Mean = 507.9
Std = 882.0
Var = 777960.4
IRI 6 Parametric Map Histograms
TTP Histogram
Mean = 181.2
Std = 208.3
Var = 43375.0
AUC Histogram
Mean = 159998.6
Std = 383751.3
Var = 147265085453.4
AUrC Histogram
Mean = 3771.9
Std = 3389.9
Var = 11491451.4
AUfC Histogram
Mean = 206246.3
Std = 382616.2
Var = 216395191451.3
Peak Intensity Histogram
Mean = 150.3
Std = 271.1
Var = 73474.6
Probability
TTP (a.u.)
TTP (Sec)
AUC (a.u.)
AUrC (a.u.)
AUfC (a.u.)
Peak Intensity (a.u.)

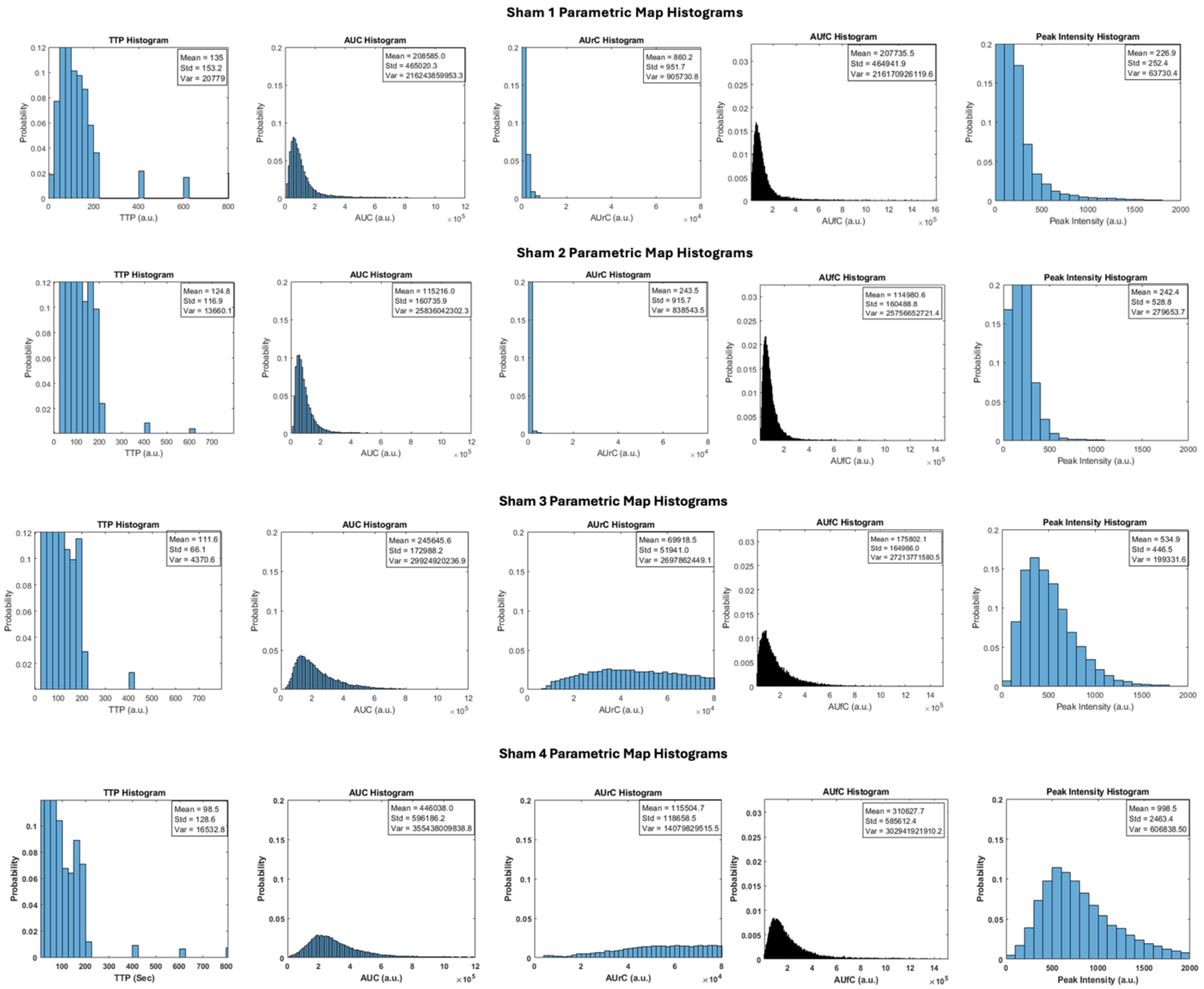

Supplementary Figure 4: Parametric mapping and histogram analysis of CEUS-derived metrics in individual kidneys. *(A) Pixel-wise parametric maps of CEUS time-intensity curve (TIC) metrics—including time-to-peak (TTP), area under the curve (AUC), area under the rising curve (AUrC), area under the falling curve (AUfC), and peak intensity (PI)—for each remaining kidney in the IRI (n=5) and sham (n=3) groups. These maps illustrate the spatial distribution and heterogeneity of contrast kinetics within the kidney.* ***(B)*** *Histograms of the corresponding pixel-wise values from each parametric map are shown for individual animals IRI (n=6) and sham (n=4). Compared to sham, IRI kidneys exhibit greater variance in AUC, AUfC, and TTP, reflecting increased heterogeneity in perfusion and retention patterns following injury. Mean and variance are provided within each plot to facilitate comparison.*

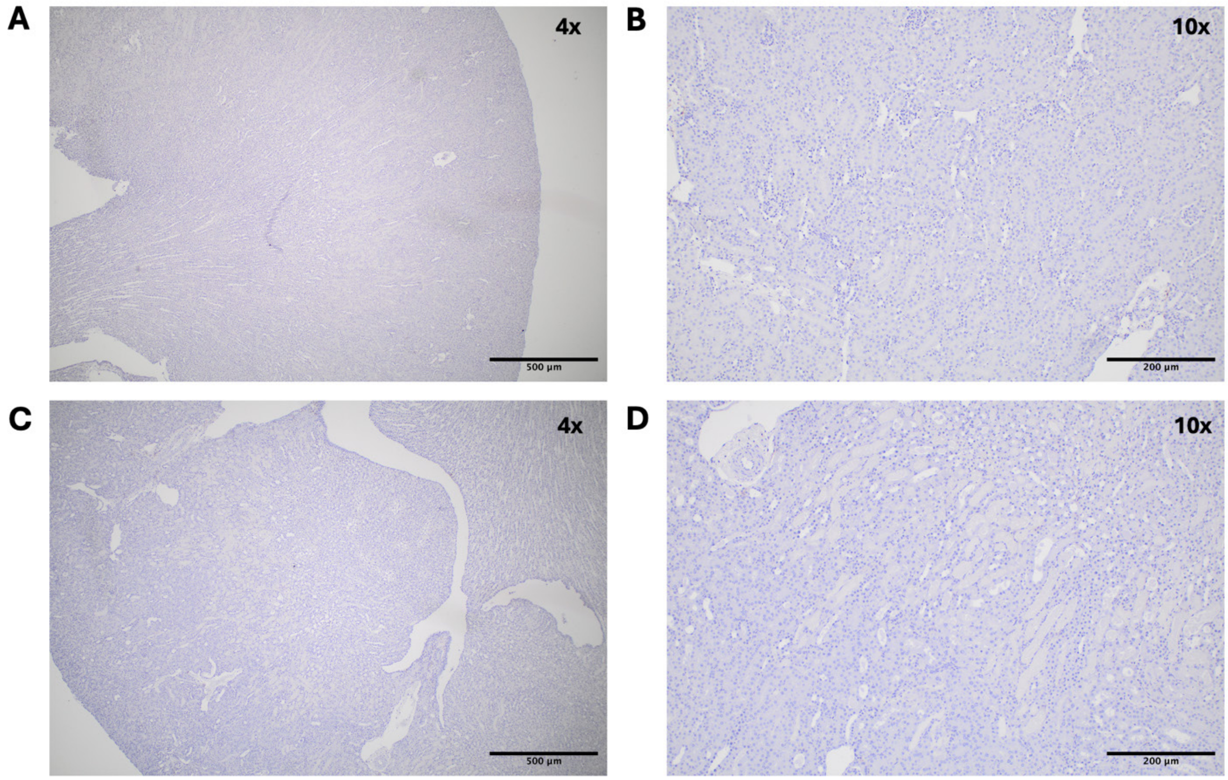

Supplementary Figure 5: F4/80 immunohistochemical staining reveals no macrophage infiltration in both Sham and IRI kidneys. *(A, B) Representative 4X and 10X magnification images of Sham kidney sections stained for F4/80, a marker of macrophages, showing* an absence of F4/80-positive staining. *(C, D) Corresponding images from IRI kidneys also show an absence of F4/80-positive cells, suggesting negligible macrophage presence at the 24-hour timepoint post-injury. Scale bars: (A, C) 500 µm; (B, D) 200 µm.*

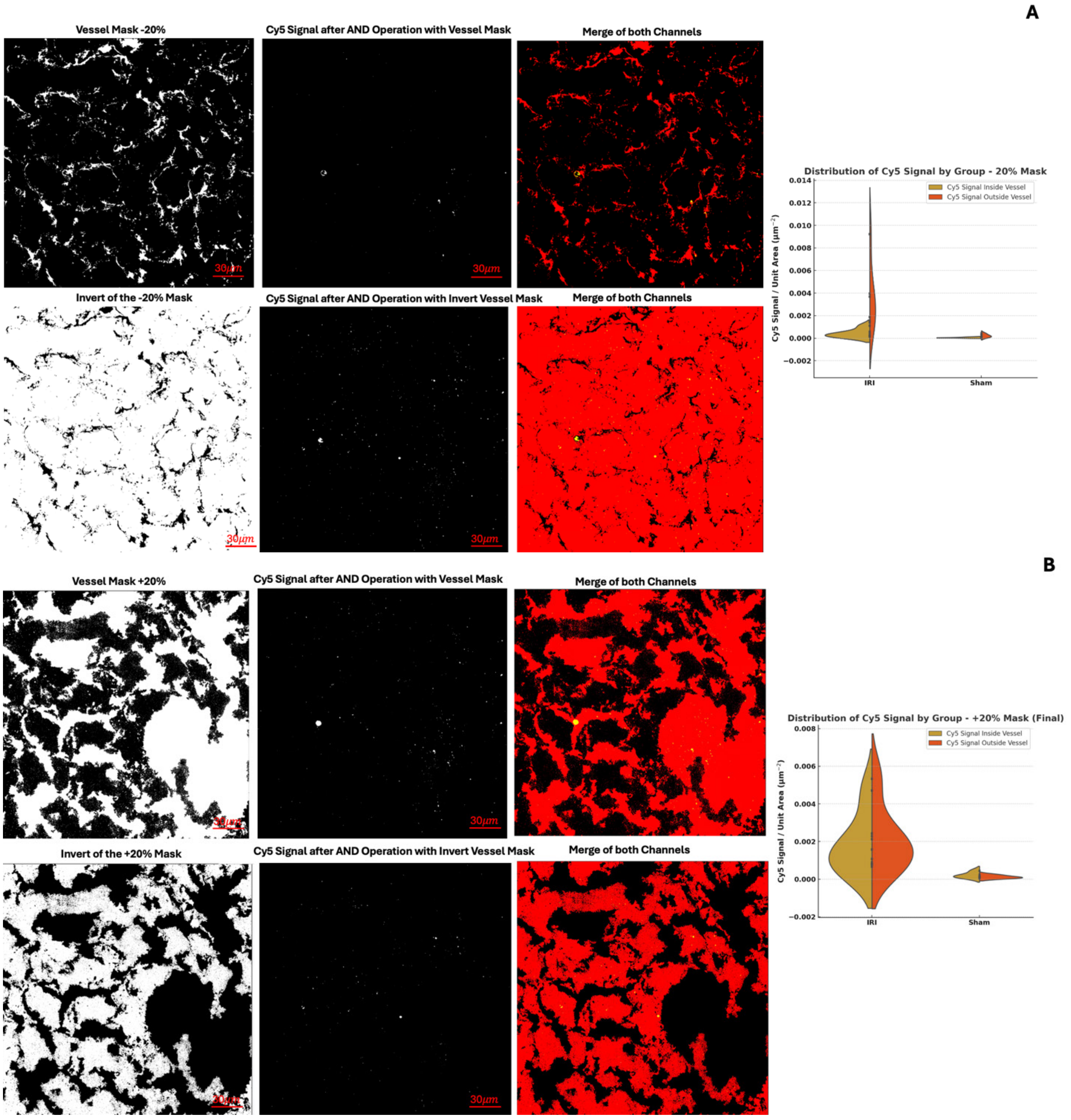

Supplementary Figure 6: Robustness of Cy5 signal distribution analysis to vessel mask variation. (A) *Example of Cy5 signal segmentation using a vessel mask threshold decreased by 20%. Top row shows the vessel mask, resulting intravascular Cy5 signal (via logical AND), and merged channels. Bottom row shows the inverted mask to extract the extravascular signal and the corresponding merged result. Violin plots summarize the Cy5 signal distribution inside and outside vessels for IRI and sham groups.* ***(B)*** *Corresponding analysis with the vessel mask threshold increased by 20%. Despite variations in vessel segmentation thresholds, the overall trend remains consistent: IRI kidneys show elevated extravascular Cy5 signal, suggestive of greater nanobubble extravasation and tissue retention. Scale bars = 30 μm.*

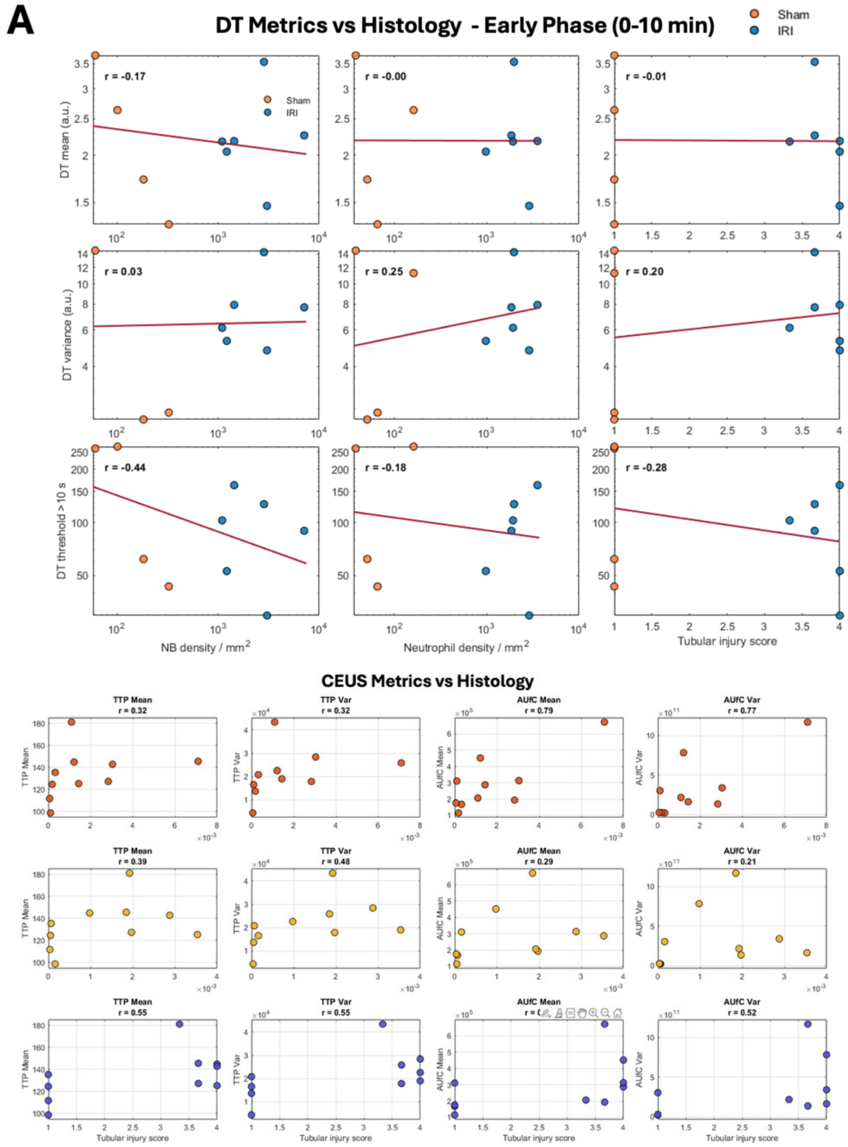

Supplementary Figure 7: **Early phase Pearson Rank Correlations Between Imaging Metrics and Histological Markers of Kidney Inflammation.** (A) Pearson rank correlation plots between early-phase (0–10 min) decorrelation time (DT) metrics, mean DT, DT variance, and percentage of pixels with DT > 10 s and histological indicators: extravasated Cy5-labeled nanobubble (NB) signal, neutrophil density ($GR1^+$ cells/μm) and tubular injury score. (B) Correlations between CEUS-derived time-to-peak (TTP) and area under the falling curve (AUfC) metrics and the same histological markers.
